# N_2_H_2_ binding to the nitrogenase FeMo cluster studied by QM/MM methods

**DOI:** 10.1007/s00775-020-01780-5

**Published:** 2020-04-07

**Authors:** Lili Cao, Ulf Ryde

**Affiliations:** Department of Theoretical Chemistry, Chemical Centre, Lund University, P. O. Box 124, 221 00 Lund, Sweden

**Keywords:** Nitrogenase, QM/MM, Broken-symmetry DFT, E_4_ state, N_2_ binding

## Abstract

We have made a systematic combined quantum mechanical and molecular mechanical (QM/MM) investigation of possible structures of the N_2_ bound state of nitrogenase. We assume that N_2_ is immediately protonated to a N_2_H_2_ state, thereby avoiding the problem of determining the position of the protons in the cluster. We have systematically studied both end-on and side-on structures, as well as both HNNH and NNH_2_ states. Our results indicate that the binding of N_2_H_2_ is determined more by interactions and steric clashes with the surrounding protein than by the intrinsic preferences of the ligand and the cluster. The best binding mode with both the TPSS and B3LYP density-functional theory methods has trans-HNNH terminally bound to Fe2. It is stabilised by stacking of the substrate with His-195 and Ser-278. However, several other structures come rather close in energy (within 3–35 kJ/mol) at least in some calculations: The corresponding cis-HNNH structure terminally bound to Fe2 is second best with B3LYP. A structure with HNNH_2_ terminally bound to Fe6 is second most stable with TPSS (where the third proton is transferred to the substrate from the homocitrate ligand). Structures with trans-HNNH, bound to Fe4 or Fe6, or cis-HNNH bound to Fe6 are also rather stable. Finally, with the TPSS functional, a structure with cis-HNNH side-on binding to the Fe3–Fe4–Fe5–Fe7 face of the cluster is also rather low in energy, but all side-on structures are strongly disfavoured by the B3LYP method.

**Electronic supplementary material:**

The online version of this article (10.1007/s00775-020-01780-5) contains supplementary material, which is available to authorized users.

## Introduction

The atmosphere of the earth contains 78% N_2_, but nitrogen is still a limiting element for plant growth. The reason for this is the strong triple bond in N_2_, which makes it highly inert. There is only one enzyme that can cleave this bond, nitrogenase (EC 1.18/19.6.1), which is present in a few bacteria and archaea [1–3]. The nitrogenases catalyse the chemical reaction.

1 $${\text{N}}_{2} + 8e^{-} + 8{\text{H}}^{ + } + 16{\text{ ATP}} \to 2{\text{NH}}_{3} + {\text{H}}_{2} + 16{\text{ADP}} + 16{\text{P}}_{i}$$

The electrons are provided by the Fe protein, which contains a [4Fe4S] cluster and also binds and hydrolyses ATP [1–3]. The Fe protein binds to the MoFe protein, which contains the P-cluster, used for electron transfer, and the catalytic FeMo cluster [4–8]. The latter is a complicated MoFe_7_S_9_C(homocitrate) cluster, although in some proteins, the Mo ion is replaced by V or Fe [9].

The nitrogenase reaction has been extensively studied with various spectroscopic and kinetic methods [1–3]. The reaction is normally described by nine states, E_0_–E_8_, differing in the number of delivered electrons (and probably also protons) [10]. It is currently believed that it is the E_4_ state that binds N_2_ and that one molecule of H_2_ dissociates in this binding process [1–3]. ENDOR experiments indicate that E_4_ contains two hydride ions that bridge between two pairs of Fe ions [3, 11, 12].

Many computational studies have also been published with the hope of giving a detailed atomistic and energetic picture of the nitrogenase reaction [3, 13–33]. Unfortunately, they have not reached any consensus so far. On the contrary, different studies have suggested either sequential (distal) or alternative protonation of N_2_ (i.e. that the first three protons are first added to one of the nitrogen atoms, which dissociates as NH_3_ before the second nitrogen atom is protonated, or the protons are added alternatively to the two nitrogen atoms so that HNNH and H_2_NNH_2_ become intermediates) [14, 15, 17, 18]. Likewise, it has been suggested that N_2_ binds either end-on or side-on and the binding can be to either one or several metals [14, 15, 20, 33]. Some groups suggest that N_2_ binds instead to the central carbide ion [17, 18]. There is not even any agreement regarding the structure of E_4_ or where protons are added beyond the E_1_ state [16, 20, 23, 25, 34].

We have recently shown that a prime problem of the computational studies is that different density functional theory (DFT) methods give widely different results [23, 25]. In particular, hybrid functionals prefer protonation of the central carbide ion, whereas pure functionals prefer the formation of metal-bound hydride ions. Therefore, the former methods suggest that E_4_ contains a triply protonated carbide ion [16, 35, 36], whereas the latter functionals suggest that it instead involves two or three hydride ions [20, 23, 25, 26]. The latter methods give results that agree better with the experimental data [3, 11, 12] and they also give geometries of the resting state closer to the crystal structure and a correct spin state on the Mo ion [25]. On the other hand, hybrid functionals seem to give better H_2_ binding energies [25].

In a series of publications, we have performed a systematic study of the reaction mechanism of nitrogenase [21–23, 25, 26]. We have used the combined quantum mechanics and molecular mechanics (QM/MM) approach [37, 38], in which the whole MoFe protein is included in the calculations. Moreover, we have tried to systematically address the problems involved in the computational study of nitrogenase, including the effect of the DFT functional, the basis set, the surroundings and the broken-symmetry state. Our aim is to systematically go through all possibilities or at least to employ some well-defined heuristic approach when the number of alternatives becomes too large. The working hypothesis is that the structures with the lowest QM/MM energy are those involved in the reaction mechanism, as it has also strongly been argued by Siegbahn [35, 39]. We have suggested structures of the E_0_–E_4_ states and shown that the previously suggested protonation states, are often quite high in energy [23, 26]. Here, we continue this investigation by studying the binding of N_2_ to the cluster.

As discussed before [23, 34], this is a formidable task: There are more than 50 possible positions where protons can bind to the FeMo cluster. This gives more than 6 million structures of the E_4_ state (50^4^) and for each there are at least 35 possible broken-symmetry states. N_2_ can also bind to a large number of sites (we tested ~ 60 structures in this study). This would give a very large number of possible structures to test (on the order of 10^10^), which is out of the reach of today’s computational resources. Fortunately, the problem can be strongly simplified. First, it is believed that H_2_ dissociates when N_2_ binds [3]. This removes two protons and two electrons, i.e. bringing the FeMo cluster to the same redox and protonation level as in the E_2_ state, for which there are “only” around 2500 possible structures. Second, it is normally assumed that the two first protonations of the N_2_ substrate take place directly after the binding [3, 40]. Again, this consumes two protons and two electrons, bringing the FeMo cluster to the same redox level as in the resting E_0_ state, with no extra protons. The structure of the resting state is known from several crystal structures [4–8].

Therefore, we study here systematically the binding of either HNNH (diazene) or NNH_2_ to the FeMo cluster of nitrogenase with QM/MM methods. Once this structure is found, it may be possible to work backwards to find possible structures also of the N_2_-bound conformation and the E_4_ state.

## Methods

### The protein

All calculations were based on the 1.0-Å crystal structure of nitrogenase from *Azotobacter vinelandii* (PDB code 3U7Q) [6]. The setup of the protein is identical to that of our previous studies of the protein [21–23, 25, 26]. The entire heterotetramer was included in the calculations, because the four subunits are entangled without any natural way to separate them. The QM calculations were concentrated on the FeMo clusters in the C subunit because there is a buried imidazole molecule from the solvent rather close to the active site (~ 11 Å) in the A subunit. The P-clusters and the FeMo cluster in subunit A were modelled by MM in the fully reduced and resting states, respectively [21].

The protonation states of all residues were the same as before [21]: all Arg, Lys, Asp, and Glu residues were assumed to be charged, except Glu-153, 440, and 231D (a letter “D” after the residue number indicates that it belongs to that subunit; if no letter is given, it belongs to subunit C; subunits A and B are identical to the C and D residues). Cys residues coordinating to Fe ions were assumed to be deprotonated. His-274, 451, 297D, 359D and 519D were assumed to be protonated on the ND1 atom, His-31, 196, 285, 383, 90D, 185D, 363D and 457D were presumed to be protonated on both the ND1 and NE2 atoms (and therefore positively charged), whereas the remaining 14 His residues were modelled with a proton on the NE2 atom. The homocitrate was modelled in the singly protonated state with a proton shared between the hydroxyl group (which coordinates to Mo) and the O1 carboxylate atom. This protonation state was found to be the most stable one in an extensive QM/MM, molecular dynamics and quantum-refinement study [21] and this protonation state is also supported by another study [41].

The protein was solvated in a sphere with a radius of 65 Å around the geometrical centre of the protein. 160 Cl^–^ and 182 Na^+^ ions were added at random positions (not inside the protein [21]) to neutralise the protein and give an ionic strength of 0.2 M [42]. The added protons, counter ions and water molecules were optimised by a simulated annealing calculation (up to 370 K), followed by a minimisation, keeping the other atoms fixed at the crystal-structure positions [21].

All MM calculations were performed with the Amber software [43]. For the protein, we used the Amber ff14SB force field [44] and water molecules were described by the TIP3P model [45]. For the metal sites, the MM parameters were the same as in our previous investigation [26]. The metal sites [21, 26] were treated by a non-bonded model [46] and charges were obtained with the restrained electrostatic potential method, obtained at the TPSS/def2-SV(P) level of theory [47, 48] and sampled with the Merz–Kollman scheme [49].

### QM calculations

All QM calculations were performed with the Turbomole software (versions 7.1 and 7.2) [50]. We employed two DFT methods, TPSS [47] and B3LYP [51–53], and two different basis sets of increasing size, def2-SV(P) [48] and def2-TZVPD [54]. The calculations were sped up by expanding the Coulomb interactions in an auxiliary basis set, the resolution-of-identity (RI) approximation [55, 56]. Empirical dispersion corrections were included with the DFT-D3 approach [57] and Becke–Johnson damping [58], as implemented in Turbomole.

**Fig. 1:**
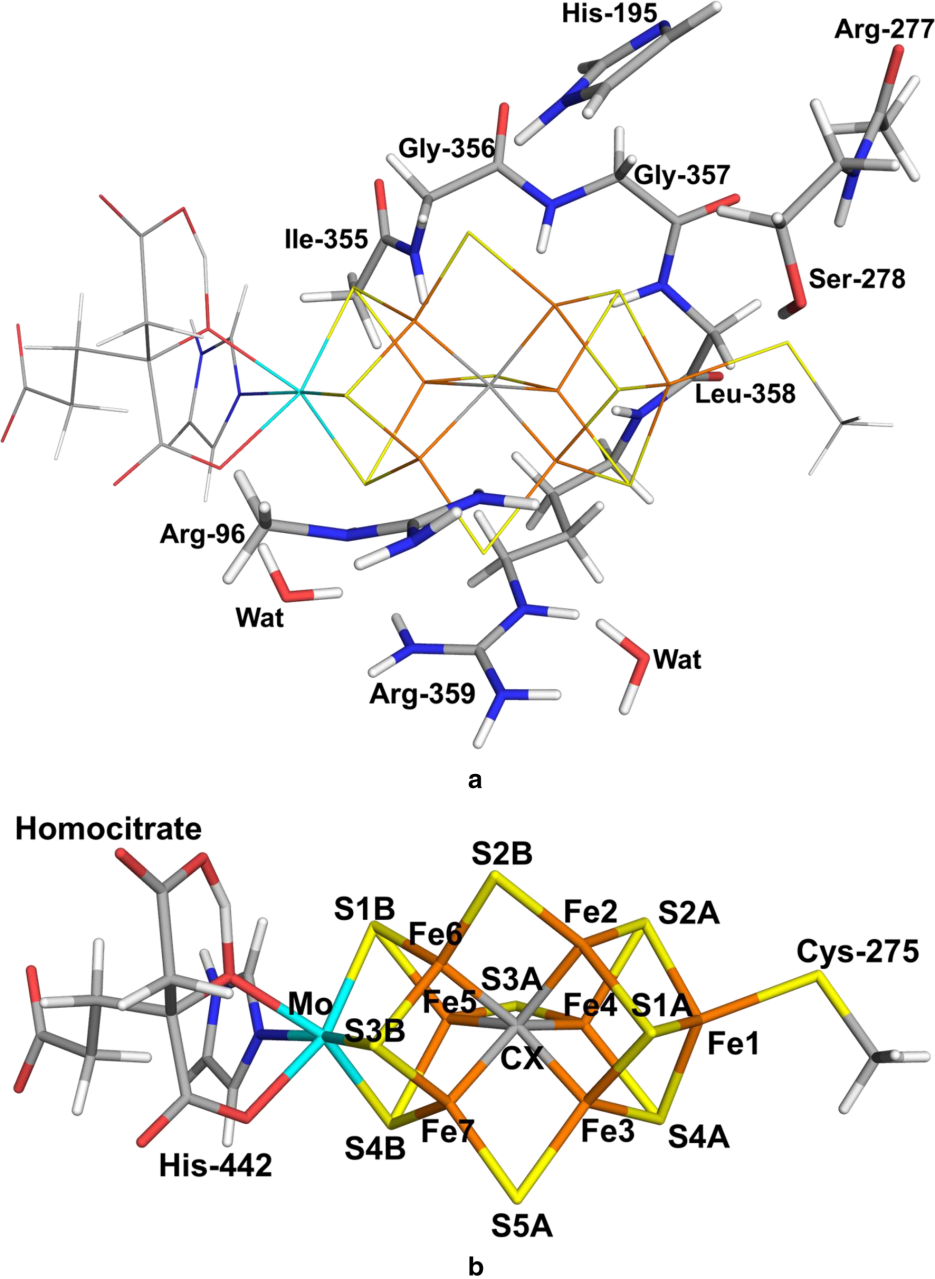
**a **The quantum system with second-sphere residues marked and **b** the FeMo cluster with atom names indicated. The view of the cluster is the same as in all other figures in this article

The FeMo cluster was modelled by MoFe_7_S_9_C(homocitrate)(CH_3_S)(imidazole), where the two last groups are models of Cys-275 and His-442. In addition, all groups that form hydrogen bonds to the FeMo cluster in the crystal structure [6] were also included, viz. Arg-96 and His-195 (sidechains), Ser-278 and Arg-359 (both backbone and sidechain, including the Cα and C and O atoms from Arg-277), Gly-356, Gly-357 and Leu-358 (backbones including the Cα and C and O atoms from Ile-355), as well as two water molecules. Moreover, all models included either HNNH or NNH_2_ binding to the cluster, giving a total of 151 atoms (shown in Fig. 1a). Following extensive Mössbauer, anomalous dispersion and QM investigations [16, 19, 41, 59], we used the oxidation-state assignment ${\text{Mo}}^{{{\text{III}}}} {\text{Fe}}_{3}^{{{\text{II}}}} {\text{Fe}}_{4}^{{{\text{III}}}}$ of the metal ions, as in the resting state, giving a net charge of – 3 for the QM system.

Experiments have shown that the ground spin state of E_0_ is quartet with a surplus of three α electrons [3, 19]. Consequently, we used this spin state for this work (previous studies have shown that structures and energies obtained with different spin are similar and that DFT calculations are not accurate enough to decide the most stable spin state [23]). The electronic structure of all QM calculations was obtained from the fragment approach by Szilagyi and Winslow to obtain a starting BS state [60]. Each of the seven Fe ions were modelled in the high-spin state, with either a surplus of α (four Fe ions) or β (three Fe ions) spin to reach the desired spin state. Such a state can be selected in 35 different ways ($\frac{7!}{{3!4!}}$) [22]. The other BS states were obtained by simply swapping the coordinates of the Fe ions [61].

We have thoroughly studied the 35 BS states for several binding modes and how their energies vary with the QM method, the size of the basis set, the geometry and the influence of the surroundings [22, 23]. The conclusion was that the effects of the basis set and the surroundings were restricted (up to 7–11 kJ/mol), the effect of geometry intermediate (up to 37 kJ/mol, but the correlation, *R*^2^, was 0.92–0.98) and that the effect of the DFT functional (TPSS or B3LYP) was large (up to 58 kJ/mol). Therefore, we first studied all systems with the same BS state (although it changed during the geometry optimisation for some states). For the best N_2_H_2_ binding modes, we performed a systematic study of all 35 BS states (obtained by simply swapping the Fe ions [22, 61]) with both the TPSS-D3 or B3LYP-D3 methods with the def2-SV(P) basis set, using optimised structures. If the BS calculations did not lead to the expected state, we assumed that this BS state is high in energy and it was not further studied. The various BS states are named by giving their number in the Noodleman nomenclature (BS1–10) [30], followed by the numbers of the three Fe ions with minority spin [26, 62], e.g. BS7-346, indicating that Fe3, Fe4 and Fe6 have β spin (the latter three numbers unambiguously define the state; the numbering of the Fe ions is taken from the 3U7Q crystal structure [6] and is shown in Fig. 1b).

For the free N_2_H_2_ ligands, the QM system was immersed into a continuum solvent, employing the conductor-like screening model (COSMO) [63, 64], implemented in Turbomole. The default optimised COSMO radii were employed and a water solvent radius of 1.3 Å [65], whereas a radius of 2.0 Å was used for the metals [66]. The dielectric constant was 80 (water).

### QM/MM calculations

The QM/MM calculations were performed with the ComQum software [67, 68]. In this approach, the protein and solvent are split into three subsystems: System 1 (the QM region) was relaxed by QM methods. System 2 contained all residues and water molecules with at least one atom within 6 Å of any atom in system 1 and it was optionally relaxed by MM. Thus, it included all atoms in residues 59, 61, 62, 65–74, 92, 95–98, 191–199, 226–231, 234, 235, 253–255, 273–282, 300, 353–355, 358–364, 377–383, 385, 386, 401 422–427, 438, 440–444, 450 and 450 from subunit C and residues 97, 98, 101 and 105 from subunit D, in total 87 residues and 35 water molecules). Finally, system 3 contained the remaining part of the protein and the solvent and it was kept fixed at the original coordinates (equilibrated crystal structure). The total system was spherical and non-periodic with 133 919 atoms.

In the QM calculations, system 1 was represented by a wavefunction, whereas all the other atoms were represented by an array of partial point charges, one for each atom, taken from the MM setup. Thereby, the polarisation of the QM system by the surroundings is included in a self-consistent manner (electrostatic embedding). When there is a bond between systems 1 and 2 (a junction), the hydrogen link-atom approach was employed: the QM system was capped with hydrogen atoms (hydrogen link atoms, HL), the positions of which are linearly related to the corresponding carbon atoms (carbon link atoms, CL) in the full system [67, 69]. All atoms were included in the point-charge model, except the CL atoms [70].

The total QM/MM energy in ComQum was calculated as [67, 68]

2 $$E_{{\text{QM/MM}}} = E_{{\text{QM1+ptch23}}}^{{{\text{HL}}}} + E_{{{\text{MM123,q}}_{{1}} { = 0}}}^{{{\text{CL}}}} - E_{{{\text{MM1,q}}_{{1}} { = 0}}}^{{{\text{HL}}}}$$

where $E_{{\text{QM1+ptch23}}}^{{{\text{HL}}}}$ is the QM energy of the QM system truncated by HL atoms and embedded in the set of point-charge modelling systems 2 and 3 (but excluding the self-energy of the point charges). $E_{{{\text{MM1,q}}_{{1}} = 0}}^{{{\text{HL}}}}$ is the MM energy of the QM system, still truncated by HL atoms, but without any electrostatic interactions. Finally, $E_{{{\text{MM123,q}}_{{1}} { = 0}}}^{{{\text{CL}}}}$ is the classical energy of all atoms in the system with CL atoms and with the charges of the QM region set to zero (to avoid double-counting of the electrostatic interactions). Thus, ComQum employs a subtractive scheme with electrostatic embedding and van der Waals link-atom corrections [71]. No cutoff is used for any of the interactions in the three energy terms in Eq. 2.

The geometry optimisations were continued until the energy change between two iterations was less than 2.6 J/mol (10^–6^ a.u.) and the maximum norm of the Cartesian gradients was below 10^–3^ a.u. For all structures, the QM/MM geometry optimisations were performed using both TPSS-D3 and B3LYP-D3 methods [47, 57] with the def2-SV(P) [48] basis set. Single-point QM/MM energies were calculated also at the TPSS-D3/def2-TZVPD level.

## Result and discussion

In this paper, we study the binding of N_2_H_2_ (we use N_2_H_2_ as a common name of both HNNH and NNH_2_) to the FeMo cofactor in nitrogenase with QM/MM methods. All structures were optimised with QM/MM, using the TPSS-D3/def2-SV(P) method. All calculations were performed for the quartet spin state, which is the observed spin state for E_0_ [3]. For the successful optimisations (i.e. leading to the desired structure), we calculated also single-point TPSS-D3 energies with the large def2-TZVPD basis set and reoptimised the structures with the B3LYP-D3/def2-SV(P) approach, because we have found very large differences between the results of pure and hybrid functionals both in this and previous studies [22, 23, 25]. We also repeated the TPSS QM/MM geometry optimisations with the MM system free to relax.

In a recent article, we examined the problem of the BS states and designed a procedure to deal with the BS states [22]. In this study, we do a similar investigation: all calculations were first studied in the BS7-235 state, which is the most stable state for the resting E_0_ state [22] and also for some of the protonated and reduced states [23], but in some cases, the BS state changed during the geometry optimisation. For all N_2_H_2_ binding modes within 20 kJ/mol of the best one (and also several other interesting states), we studied all possible BS states. For simplicity, we discuss only the results obtained with the best BS state, whereas in the tables, results are given also for the BS7-235 state, if available.

Below, we describe the obtained structures for end-on NNH_2_, side-on HNNH, as well as end-on HNNH in separate sections. We discuss first the results obtained with the TPSS functional and a fixed protein surrounding outside the QM system. Finally, we discuss how the results change when the surrounding protein is allowed to relax or when the functional is changed to B3LYP.

### End-on binding of NNH2

To begin with, we studied end-on binding modes of NNH_2_. We tested both terminal binding to a single Fe ion, bridging between pairs of Fe ions, or binding to four Fe ions. However, we ignored binding to the Fe1 and Mo atoms, because such structures were high in energy in our previous work [23] (we checked some cases in this project to confirm that it is true also for N_2_H_2_) and Hoffman and coworkers have argued against N_2_ binding to Mo [3, 72]. For the terminal binding, we tested only one conformation, viz. trans to the central carbide ion, because such structures were most stable for hydride binding [23] and because the NNH_2_ molecule is quite large, so there is not much room for alternative binding modes in the protein structure. When NNH_2_ binds to two Fe ions belonging to the same subcluster (e.g. Fe2–Fe4 or Fe5–Fe7), only one conformation is possible, giving rise to 3 + 3 = 6 possible structures, as shown in Table 1. Each structure is named by giving the number of the metal ions to which the N atoms bind, e.g. Fe2/4, indicating that the N atom binds to both Fe2 and Fe4 (the numbering of the Fe ions is shown in Fig. 1b). However, when NNH_2_ bridges Fe ions on different sides of the cluster, Fe2/6, Fe3/7 and Fe4/5, two conformations are possible, depending on which side of the belt (µ_2_) sulfide ions they are located. These two conformations are named after which belt sulfide atom they are directed towards, e.g. Fe2/6(3) or Fe2/6(5), indicating that NNH_2_ is on the side of the S2B ion that is directed towards S3A or S5A, respectively (again the names of the sulfide ions are taken from the crystal structure [6] and are shown in Fig. 1b). Finally, for the binding to four Fe ions, there is only a single conformation for each face of the cluster, which we call Fe2/3/6/7, Fe3/4/5/7 and Fe2/4/5/6, indicating the four Fe ions involved. This gives 21 possible structures, shown in Table 1.

**Table 1:** Binding modes of N_2_H_2_ tested

End-on NNH2	Side-on cis-HNNH
Fe2	Fe2Fe2
Fe3	Fe3Fe3
Fe4	Fe4Fe4
Fe5	Fe5Fe5
Fe6	Fe6Fe6
Fe7	Fe7Fe7
Fe2/3	Fe2Fe3
Fe2/4	Fe2Fe4
Fe3/4	Fe3Fe4
Fe5/6	Fe5Fe6
Fe5/7	Fe5Fe7
Fe6/7	Fe6Fe7
Fe2/6(3)	Fe2Fe6(3)
Fe2/6(5)	Fe2Fe6(5)
Fe3/7(2)	Fe3Fe7(2)
Fe3/7(3)	Fe3Fe7(3)
Fe4/5(2)	Fe4Fe5(2)
Fe4/5(5)	Fe4Fe5(5)
Fe2/3/6/7	Fe2/3Fe6/7
	Fe2/6Fe3/7
Fe2/4/5/6	Fe2/4Fe5/6
	Fe2/6Fe4/5
Fe3/4/5/7	Fe3/4Fe5/7
	Fe3/7Fe4/5
	Fe2Fe7
	Fe3Fe6
	Fe4Fe6
	Fe2Fe5
	Fe4Fe7
	Fe3Fe5

The binding of each N atom is indicated by “Fe” and the number of the ion it binds to. A slash (/) indicates that a N atom binds to several metal ions. All side-on binding modes to two Fe ions had the Fe–Fe and N–N vectors parallel; the FeMo cluster turned out to be too crowded to allow for any transverse side-on binding modes

In many cases, the optimisation failed to give the desired structures, e.g. because the structure reorganised to another structure or the protein structure is too crowded. If so, we tried to obtain it using one or two N–Fe distance restraints and if the geometry optimisation was successful, the restraints were removed and the structures were reoptimised (thus, all presented structures were obtained without any restraints). If this did not give the desired structure, we made no further attempts to get it.

**Fig. 2:**
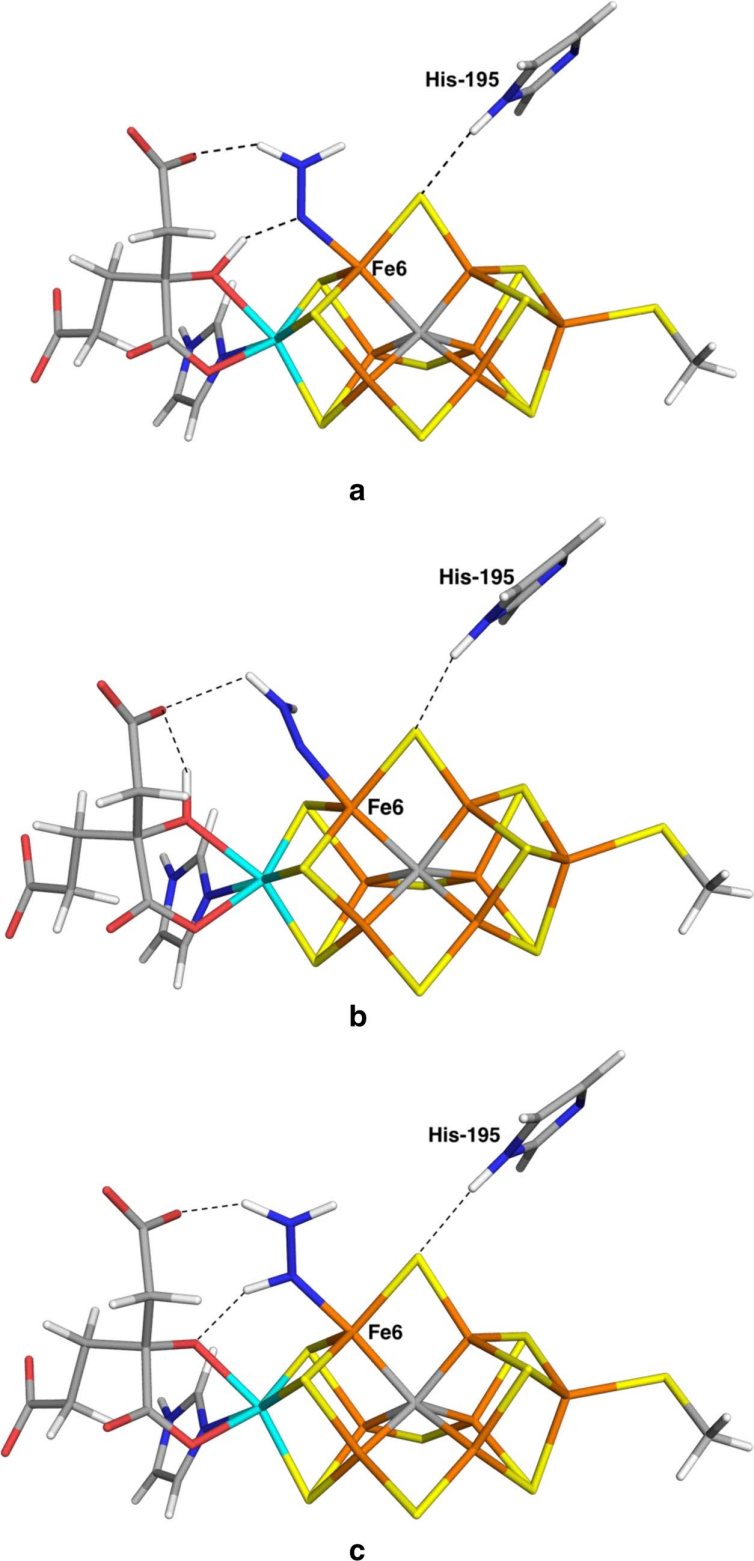
The most stable structures for end-on binding of NNH_2_, obtained at the TPSS/def2-SV(P) level of theory: **a** Fe6(HCA), **b** Fe6 and **c** Fe6(HNNH_2_). The Fe, Mo, S, C, N, O and H atoms are orange, cyan, yellow, grey, blue, red and white, respectively. Hydrogen bonds to homocitrate and His-195 are indicated with broken lines

The results of the optimisations are shown in Tables 2 (energies) and 3 (structures). It can be seen that we managed to obtain 19 of the structures. The most favourable binding mode was to Fe6. We found four different structures with NNH_2_ binding end-on to Fe6. One structure was stabilised by a hydrogen bond between the N atom that binds to Fe6 and the alcoholic proton of homocitrate (HCA; 1.56 Å; Fig. 2a). Consequently, we call this structure Fe6(HCA). Moreover, one of the two protons on NNH_2_ forms another hydrogen bond to the acetate group of homocitrate (which normally forms an internal hydrogen bond to the alcohol group; 1.60 Å). It had Fe6–N and N–N bond lengths of 1.85 and 1.26 Å. The latter is somewhat longer than in free NNH_2_, 1.21 Å, calculated at the same level of theory. The Mulliken spin population on Fe6, 1.6, is appreciably smaller than those on other iron ions, 2.5–3.2 (in absolute terms; shown in Table S1). For this structure, we studied the relative stabilities of all possible BS states and found that BS10-135 was most stable (Table S2), 8–23 kJ/mol more stable than BS7-235 with both functionals and basis sets. The Fe6–N bond length increased by 0.03 Å when going from BS7-235 to BS10-135 and the spin population on Fe6 increased by 0.5.

**Table 2:** Relative energies of the various structures obtained

Structure	BS	TP	TZ	Free	B3	B3Free
End-on NNH_2_ binding						
Fe1	BS7-235	111	93	100	120	
Fe2	BS7-235	115	91	135	130	
Fe3	BS10-147	107	122	132	169	
Fe4	BS7-235	163	215	209	213	
Fe5	BS7-235	178	169	179	128	
Fe6	BS10-147	56 (71)	44	91	90 (122)	70
Fe6(HCA)	BS10-135	38 (47)	28 (47)	52 (70)	68 (90)	51
Fe6(HNNH_2_)	BS10-147	9	3	23	29	50
Fe6(S2B)	BS10-127	87	61	144	138	
Fe7	BS7-235	121	130	162	118	
Fe3(S2A)	BS7-235	136	159	177	113	
Fe2/4	BS7-235	168	158	188	192	
Fe3/4	BS7-235	78	75	110	107	
Fe2/6/7	BS7-235	230	235	207	287	
Fe2/6(5)	BS10-147	183	171	185	230	
Fe3/7(2)	BS5-256	155	171	145	234	
Fe3/7(3)	BS7-235	102	143	152	182	
Fe4/5(2)	BS7-235	171	161	195	193	
Fe4/5(5)	BS7-235	77	84	114	125	
Fe2/3/6/7	BS7-235	230	235	220	287	
Fe2/4/5/6	BS7-235	233	240	225	310	
Fe3/4/5/7	BS7-235	111	116	139	216	
Side-on cis-HNNH binding						
Fe2Fe2	BS10-147	107	94	157	164	
Fe4Fe4	BS10-147	217	197	163	340	
Fe5Fe5	BS10-146	196	243	166	283	
Fe6Fe6	BS7-235	197	166	200	167	
Fe7Fe7	BS7-235	150	136	195	205	
Fe2/3Fe6/7	BS7-235	242	257	215	278	
Fe2/6Fe3/7	BS7-235	185	177	221	248	
Fe2/6Fe3/7tr^c^	BS6-156	70	90	128	257	
Fe2/4Fe5/6	BS2-234	220	211	243	296	
Fe2/6Fe4/5	BS3-134	276	269	215	413	
Fe2/6Fe4	BS7-235	225	273	178	381	
Fe3/7Fe4/5	BS2-234	40 (59)	31 (64)	85	186 (183)	192
Fe2Fe7	BS7-235	204	223	221	203	
Fe3Fe6	BS10-147	199	244	185	252	
Fe2Fe6(3)	BS10-147	115	99	161	165	
Fe2Fe6(5)	BS7-235	179	189	215	207	
Fe3/7Fe3(2)	BS8-245	117	114	155	246	
Fe3Fe7(3)	BS5-256	113	106	135	206	
Fe4/5Fe5(2)	BS7-235	156	143	207	262	
Fe4Fe5(5)	BS2-234	120	125	150	236	
End-on trans-HNNH binding						
Fe2(trans)	BS10-147	0	0	0	0^a^	0^a^
Fe3(trans)	BS10-146	69	64	90	88	
Fe4(trans)	BS7-235	29	28	63	75	35
Fe5(trans)	BS10-147	158	149	109	206	
Fe6(trans)	BS10-147	10	19	29	35^b^	62
Fe7(trans)	BS10-147	143	136	100	248	
End-on cis-HNNH binding						
MoFe6(cis)	BS7-235	176	213	215	162	
Fe2(cis)	BS10-147	17 (51)	12 (44)	86	18^a^ (36)	46
Fe3(cis)	BS10-146	116	102	134	131	
Fe4(cis)	BS7-235	84	82	107	127	
Fe5(cis)	BS7-235	124	122	132	84	
Fe6(cis)	BS10-135	48	32	34	64	51
Fe7(cis)	BS7-235	204	189	198	269	0

Up to five energies are given: TP—TPSS-D3/def2-SV(P) optimised geometries with surroundings fixed, TZ—single-point TPSS-D3/def2-TZVPD on the TP structures, Free—TPSS-D3/def2-SV(P) optimised geometries with the surroundings relaxed, B3—B3LYP-D3/def2-SV(P) optimised geometries with surroundings fixed and B3Free—B3LYP-D3/def2-SV(P)-optimised geometries with the surroundings relaxed. Column BS indicates the studied (best) BS state. If additional energies are given in brackets, they are for the BS7-235 state. In some cases, the best BS state is different for TPSS and B3LYP; then the best B3LYP state is indicated in a footnote

^a^BS8-236

^b^BS2-267

^c^In this structure, one of the protons of HNNH has moved to S5A, whereas the ligand has taken a proton from Arg-96 (cf. Figure S2)

**Table 3:** Geometries of the various structures obtained

Structure	BS	TPSS/def2-SV(P) fixed protein					TPSS/def2-SV(P) relaxed protein					B3LYP/def2-SV(P)				
		N	Fe	Fe	Fe	Fe/other	N	Fe	Fe	Fe	Fe/other	N	Fe	Fe	Fe	Fe/other
End-on NNH_2_ binding																
Fe1	BS7-235	1.27	1.79				1.27	1.79				1.27	1.79			
Fe2	BS7-235	1.25	1.92				1.25	1.92				1.22	2.10			
Fe3	BS10-147	1.27	1.81				1.27	1.84				1.27	1.80			
Fe4	BS7-235	1.28	1.80				1.28	1.82				1.24	1.85			
Fe5	BS7-235	1.43	1.90			S3A = 1.78	1.43	1.94			S1A = 1.80	1.43	1.93			S3A = 1.76
Fe6	BS10-147	1.26	1.86				1.26	1.83				1.26	1.86			
Fe6(HCA)	BS10-135	1.26	1.85									1.25	1.84			
	BS7-235	1.26	1.82									1.25	1.82			
Fe6(HNNH_2_)	BS10-135	1.28	1.84									1.29	1.87			
	BS10-147	1.28	1.84				1.28	1.84				1.30	1.88			
Fe6(S2B)	BS10-127	1.38	1.84			S2B = 1.70	1.38	1.84			S2B = 1.72	1.35	1.87			S2B = 1.70
Fe7	BS7-235	1.26	1.85				1.26	1.85				1.25	1.84			
Fe3(S2A)	BS7-235	1.42	1.93			S2A = 1.73	1.41	1.95			S2A = 1.76	1.41	1.97			S2A = 1.73
Fe2/4	BS7-235	1.39	1.98	1.87		S1A = 1.76	1.39	1.95	1.89		S1A = 1.76	1.38	2.05	2.19		S1A = 1.68
Fe3/4	BS7-235	1.31	1.83	1.76			1.32	1.86	1.78			1.26	2.06	1.97		
Fe2/6/7	BS7-235	1.35	1.90	1.87	1.86		1.38	1.93	1.88	1.90		1.33	1.93	1.98	1.87	
Fe2/6(5)	BS10-147	1.39	1.88	1.87		S2B = 1.76	1.42	1.92	1.88		S2B = 1.77	1.37	1.72	1.92		S2B = 1.72
Fe3/7(2)	BS5-256	1.29	1.81	1.80			1.31	1.81	1.83			1.27	1.82	1.84		
Fe3/7(3)	BS7-235	1.31	1.90	1.86			1.32	1.91	1.86			1.25	2.02	1.86		
Fe4/5(2)	BS7-235	1.40	1.92	1.90		S3A = 1.75	1.42	1.95	1.92		S3A = 1.76	1.40	2.02	1.94		S3A = 1.72
Fe4/5(5)	BS7-235	1.28	1.81	2.18			1.29	1.81	2.20			1.27	1.81	2.21		
Fe2/3/6/7	BS7-235	1.35	1.90	2.64	1.87	1.86	1.38	1.91		1.88	1.91	1.36	1.91	2.36	2.03	1.87
Fe2/4/5/6	BS7-235	1.39	2.12	1.86	1.92	2.49	1.36	2.14	1.97	2.15	1.96	1.37	2.16	1.89	1.95	2.62
Fe3/4/5/7	BS7-235	1.35	2.43	1.93	2.07	1.96	1.35		1.92	2.02	1.97	1.33	1.93	2.22	1.90	
Side-on cis-HNNH binding																
Fe2Fe2	BS10-147	1.32	2.13		2.03		1.31	2.12		2.11		1.30	2.13		2.04	
Fe4Fe4	BS10-147	1.37	2.02		1.95		1.34	2.10		1.97		1.34	2.09		1.97	
Fe5Fe5	BS10-146	1.33	2.02		2.02		1.33	2.07		2.00		1.31	2.04		2.01	
Fe6Fe6	BS7-235	1.37	1.95		2.04	S2B = 1.76	1.38	1.95		2.04	S2B = 1.77	1.34	1.95		2.04	S2B = 1.75
Fe7Fe7	BS7-235	1.38	1.92		1.97	S5A = 1.80	1.38	1.92		1.98	S5A = 1.81	1.33	2.08		1.96	S5A = 1.78
Fe2/3Fe6/7	BS7-235	1.37	1.99	1.96	1.97	1.94	1.39	2.01	1.99	1.99	1.99	1.30	2.21	2.15	1.92	
Fe2/6Fe3/7	BS7-235	1.35	1.96	2.03	1.94	2.00	1.34	2.01	2.05	2.03	2.08	1.35	1.96	2.03	1.94	2.00
Fe2/6Fe3/7tr	BS6-156	1.40	1.94	1.93	1.92	1.93	1.39	1.94	1.94	1.92	1.93	1.33	1.96	2.16	1.91	2.02
Fe2/4Fe5/6	BS2-234	1.39	1.93	1.90	1.89	2.03	1.40	1.94	1.97	1.96	2.01	1.38	1.98		1.91	2.00
Fe2/6Fe4/5	BS3-134	1.40	1.87	1.89	1.92	1.92	1.39	1.93	1.95	1.96	1.96	1.38	1.91	1.89	1.91	1.90
Fe2/6Fe4	BS7-235	1.40	1.87	1.93	1.96		1.40	1.92	1.98	1.96		1.39	1.87	1.94	1.95	
Fe3/7Fe4/5	BS2-234	1.40	1.95	1.94	1.97	1.95	1.40	1.95	1.94	1.98	1.95	1.33	2.19	1.95	2.20	1.97
Fe2Fe7	BS7-235	1.27	2.00		1.93		1.27	2.06		1.94		1.26	2.10		1.95	
Fe3Fe6	BS10-147	1.28	1.85		1.92		1.28	1.87		1.96		1.26	1.86		1.93	
Fe2Fe6(3)	BS10-147	1.41	1.94		1.93	S2B = 1.76	1.41	1.95		1.93	S2B = 1.77	1.39	1.95		1.99	S2B = 1.75
Fe2Fe6(5)	BS7-235	1.29	1.93		1.88		1.29	1.90		1.92		1.26	2.10		1.92	
Fe3/7Fe3(2)	BS8-245	1.38	1.91	1.97	2.01		1.39	1.91	1.97	2.00		1.38	1.96	2.01	1.98	
Fe3Fe7(3)	BS5-256	1.29	1.88		1.87		1.29	1.86		1.88		1.30	1.91		1.89	
Fe45Fe5(2)	BS7-235	1.41	1.95	2.35	1.98		1.41	1.96	2.31	1.99		1.36	1.94	2.25	2.04	
Fe4Fe5(5)	BS2-234	1.29	1.82		1.87		1.29	1.87		1.82		1.29	1.83		1.90	
End-on trans-HNNH binding																
Fe2(trans)	BS10-147	1.26	1.94				1.25	1.96				1.23	2.13			
Fe3(trans)	BS10-146	1.25	1.92				1.25	1.94				1.24	1.90			
Fe4(trans)	BS7-235	1.26	1.88				1.26	1.89				1.24	1.90			
Fe5(trans)	BS10-147	1.26	1.88				1.26	1.93				1.24	1.89			
Fe6(trans)	BS10-147	1.25	1.91				1.26	1.93				1.23	2.07			
Fe7(trans)	BS10-147	1.28	2.08				1.29	2.07				1.24	2.13			
End-on cis-HNNH binding																
MoFe6(cis)	BS7-235	1.32	2.19	2.11			1.32	2.17	2.27			1.32	2.20	2.11		
Fe2(cis)	BS10-147	1.26	1.92				1.26	1.93				1.23	2.10			
Fe3(cis)	BS10-146	1.26	1.89				1.26	1.93				1.25	1.88			
Fe4(cis)	BS7-235	1.27	1.87				1.28	1.87				1.26	1.89			
Fe5(cis)	BS7-235	1.25	2.40				1.24	HNNH released				1.23		HNNH released		
Fe6(cis)	BS10-135	1.25	1.90				1.26	1.91				1.24	1.94			
Fe7(cis)	BS7-235	1.29	1.88				1.29	1.89				1.28	1.90			

Three geometries are given: TPSS-D3/def2-SV(P) optimised with surroundings fixed, TPSS-D3/def2-SV(P) optimised with surroundings relaxed, and B3LYP-D3/def2-SV(P) optimised with surroundings fixed. BS indicates the studied BS state. The “other” columns give N–S distances, if they are shorter than 2.0 Å. The Fe–N distances are given in the order indicated by the name of the structure

If the hydrogen bonds between NNH_2_ and the homocitrate ligand are replaced with the normal internal hydrogen bond within homocitrate (we call that structure simply Fe6, shown in Fig. 2b), the structure was 18 kJ/mol less stable (16 kJ/mol with the large def2-TZVPD basis set). It turned out to be more stable in the BS10-147 state, which was 51 kJ/mol more stable than the BS7-235 state.

However, if the proton is transferred from the homocitrate alcohol group to the substrate, giving HNNH_2_, still bound end-on to Fe6 [therefore called Fe6(HNNH_2_)], the structure (shown in Fig. 2c) is actually stabilised by 25–29 kJ/mol, therefore constituting the most stable structure of this type. It had Fe6–N and N–N bond lengths of 1.84 and 1.28 Å (the latter is 0.05 Å longer than in free HNNH_2_). It forms the same two hydrogen bonds with homocitrate as the Fe6(HCA) structure (although with the opposite polarity for one of them), with H–O distances of 1.69 and 1.60 Å. This structure was also found to be more stable in the BS10-147 state. The spin population was still lowest on Fe6, although it was somewhat higher, 1.8, compared to 2.5–3.2 for the other Fe ions.

Finally, we found a fourth structure, in which there is a close interaction between the N atom that binds to Fe6 and S2B (N–S distance of 1.70 Å). This corresponds to N–S bonds, reflecting that the QM calculations allow for chemical reactions. Such bonds are observed in nine of the structures in Table 3 and they are also characterised by a significantly elongated N–N bond (1.38 Å). They are not among the most stable structures [the present structure, called Fe6(S2B), is the best, 57–79 kJ/mol less stable than Fe6(HNNH_2_)], but their energies are comparable to many of the other structures without N–S bonds.

Two structures with other binding modes were more stable than the latter structure. The Fe4/5(5) binding mode was 69 kJ/mol less stable than Fe6(HNNH_2_) (81 kJ/mol with the def2-TZVPD basis set). In this structure, the N atom bridges two Fe ions from different subclusters, but it is much closer to Fe4 (1.81 Å) than to Fe5 (2.18 Å). The N–N bond length is the same as in the Fe6(HNNH_2_) binding mode, 1.28 Å. The Fe3/4 structure has a similar stability, being 69–72 kJ/mol less stable than Fe6(HNNH_2_) with the two basis sets. In this structure, the nitrogen atom bridges between Fe3 and Fe4 (i.e. Fe ions from the same subcluster) with distances of 1.83 and 1.76 Å. The N–N bond length is 1.31 Å, i.e. 0.03 Å longer than in Fe6(HNNH_2_). Interestingly, S4A has dissociated from Fe4 in this structure (3.6 Å distance, cf. Figure S1 in the supplementary material). Both structures were most stable in the BS7-235 state.

The other structures of this type are at least 93 kJ/mol less stable than Fe6(HNNH_2_). It can be seen from Table 2 that there is no clear relation between the type of structures and their energies, indicating that the stability is mainly determined by whether NNH_2_ can fit into the structure without clashing with the surrounding protein. In general, structures with NNH_2_ binding to two Fe ions give longer N–N bond lengths (1.28–1.35 Å) than those binding to one Fe ion (1.25–1.28 Å, disregarding those with close N–S interactions), and those with NNH_2_ binding to four Fe ions give even longer N–N bonds (1.35–1.39 Å).

### Side-on binding of HNNH

Next, we performed a similar investigation for complexes with side-on binding. In this case, we assumed that the substrate binds in the form of cis-HNNH, where the hydrogen atoms are on the same side of the N–N bond and therefore do not interfere with the binding. We tested side-on binding to the same Fe ion, to two or four different metal ions. Each structure is named by giving the number of the metal ions to which each of the N atoms bind, e.g. Fe2Fe2 (same metal), Fe2Fe3 (bridging two metal ions) and Fe2/3Fe6/7 (indicating that the first N atom binds to Fe2 and Fe3, whereas the second binds to Fe6 and Fe7). This way, it can be directly seen if we discuss a side-on structure (two “Fe”) or an end-on structure (one “Fe”). It can also be seen to which metal ions the various N atoms bind. All side-on binding modes to two Fe ions had the Fe–Fe and N–N vectors parallel; the FeMo cluster turned out to be too crowded to allow for any transverse side-on binding modes.

For most side-on structures, we studied only a single conformation (with the two H atoms on HNNH pointing away from the cluster. However, when HNNH bridges the closest Fe ions between the two subclusters, (Fe2Fe6, Fe3Fe7 and Fe4Fe5), two conformations are possible, depending on which side of the belt sulfide ions they are located. Again, the name reflects the direction of the group, e.g. Fe2Fe6(3) or Fe2Fe6(5), indicating that HNNH is on the same side as S3A or S5A, respectively. When HNNH bind to four Fe ions on the three faces of the FeMo cluster, two conformations are possible, depending on whether the N–N bond is parallel with or perpendicular to the approximate *C*_3_ axis of the cluster (through the Mo, Fe1 and C atoms). However, these already have different names, e.g. Fe2/3Fe6/7 and Fe2/6Fe3/7, because the N atoms bind to different Fe ions. Finally, HNNH can also bridge more distant Fe ions, diagonally over the cluster face, e.g. Fe2Fe5. In total, we tested 30 possibilities, as it is shown in Table 1.

The results of this investigation are also included in Tables 2 and 3. It can be seen that we found only 20 of the tested structures. In particular, all structures involving binding to two Fe ions within the same subcluster reorganised to other structures. Likewise, only two structures with HNNH binding diagonally between the two subclusters were found.

**Fig. 3:**
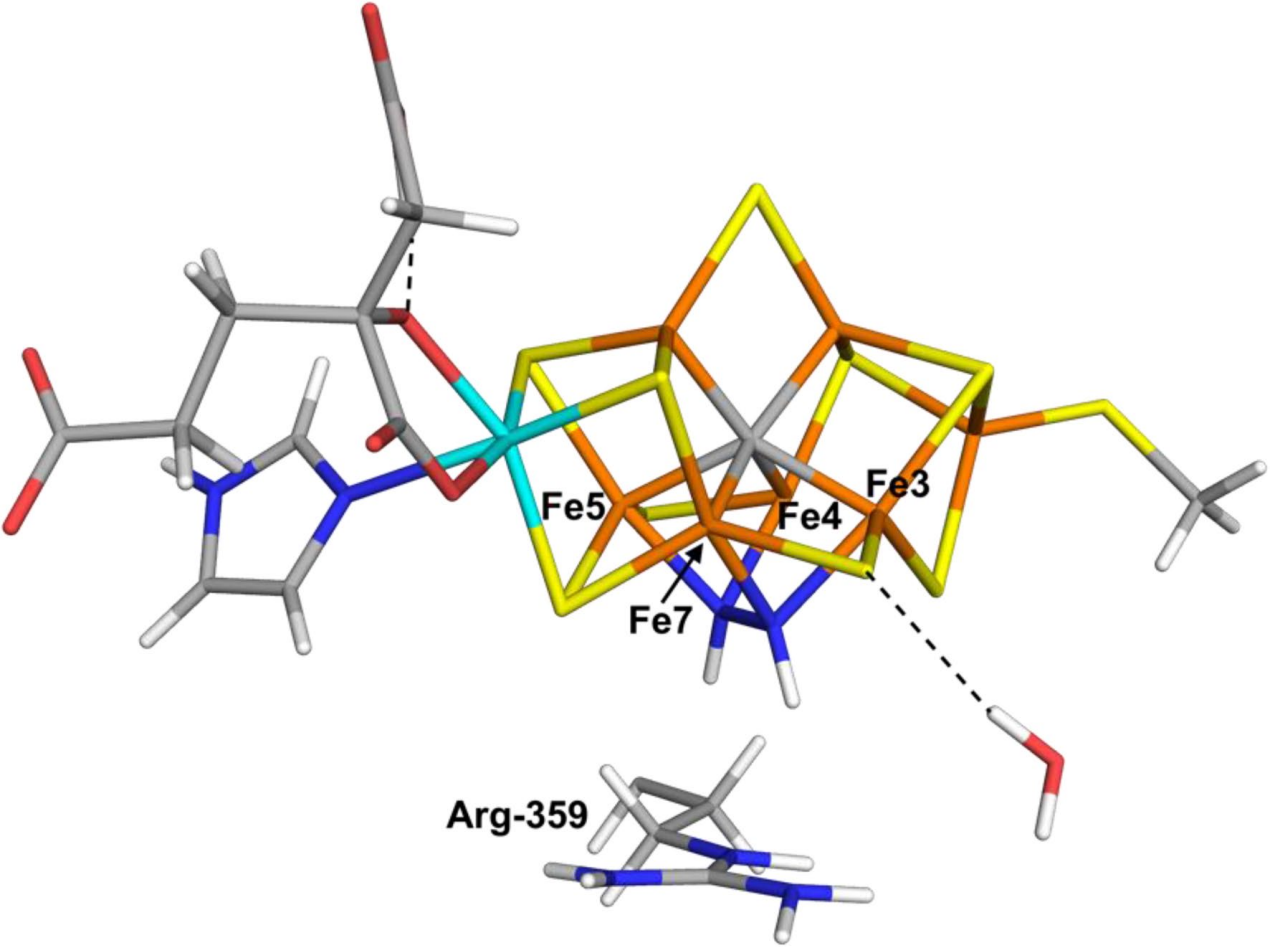
The most stable structure for side-on binding of HNNH, obtained at the TPSS/def2-SV(P) level of theory, Fe3/7Fe4/5. The water molecule is HOH-525C in the crystal structure [6]

At the TPSS level, the best binding mode is Fe3/7Fe4/5. As can be seen in Fig. 3, HNNH bridges between the Fe3/7 and Fe4/5 pairs, with the N–N bond perpendicular to the Fe1–Mo axis. All Fe–N bonds are of a similar length, 1.94–1.97 Å. The N–N bond length is 1.40 Å, i.e. appreciably longer than in free cis-HNNH (1.24 Å). This structure is 27–31 kJ/mol less stable than the Fe6(HNNH_2_) end-on binding mode with the two basis sets. An investigation of all 35 BS states showed that BS2-234 was lowest in energy, 19 kJ/mol more stable than BS7-235 (33 kJ/mol with the big basis set). It had low spin populations on all four Fe ions binding HNNH, 0.8–1.8 (in absolute terms). The spin population on the other three Fe ions was higher, but still rather low, 2.4–3.0.

The second-best side-on binding mode was similar, viz. the Fe2/6Fe3/7tr structure, where HNNH also binds to four Fe ions, although on another face of the cluster. However, this structure is peculiar in that one of the protons of HNNH has moved to S5A, whereas the ligand has taken a proton from Arg-96, forming a short hydrogen bond to this group (1.57 Å; cf. Figure S2). This structure is 30 kJ/mol less stable than the Fe3/7Fe4/5 structure (the corresponding structure without this transfer, Fe2/6Fe3/7, is 115 kJ/mol less stable). Five structures [Fe2Fe2, Fe3Fe7(3), Fe2/Fe6(3), Fe3/7Fe3(2) and Fe4Fe5(5)] were 67–80 kJ/mol less stable than Fe3/7Fe4/5, whereas the other structures were 110–236 kJ/mol less stable than Fe3/7Fe4/5. Again, structures with HNNH binding to four Fe ions gave longer N–N bond lengths (1.35–1.40 Å) than those binding to two Fe ions (1.27–1.33 Å), with the exception of the Fe4Fe4 complex (1.37 Å).

### End-on binding of HNNH

Several of the side-on structures ended up in structures in which HNNH instead bound terminally to a Fe ion. In principle, this is not unexpected, because for free N_2_H_2_, cis-HNNH is 60–73 kJ/mol more stable than NNH_2_, depending on the functional, basis set and whether the calculations are performed in vacuum or in a COSMO continuum solvent with a dielectric constant of 80 (water). Moreover, cis-HNNH is 21–27 kJ/mol less stable than trans-HNNH in vacuum, but only 11 kJ/mol less stable in the water-like continuum solvent. Therefore, we decided to study also such complexes systematically, looking for complexes with cis- or trans-HNNH terminally bound to any of the seven Fe ions and also Mo.

**Fig. 4:**
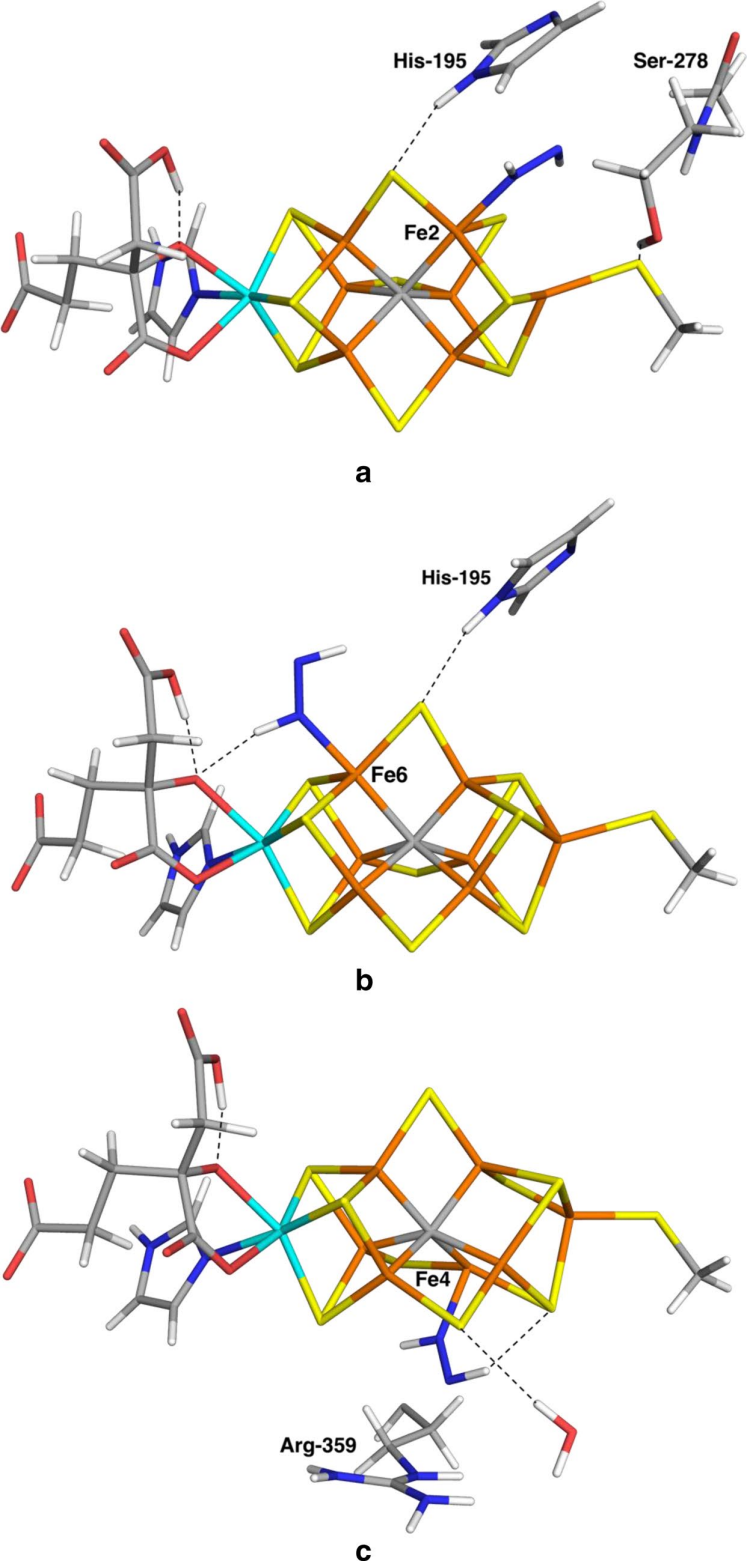
The most stable structures for end-on binding of trans-HNNH, obtained at the TPSS/def2-SV(P) level of theory, **a** Fe2(trans), **b** Fe6(trans) and **c** Fe4(trans)

For trans-HNNH, we found six complexes. Interestingly, the complex with trans-HNNH terminally bound to Fe2 [we will call it Fe2(trans) in the following to discern it to the corresponding Fe2(NNH_2_) and Fe2(cis-HNNH) complexes] turned out to be the most stable complex in this study. It has a N–N bond length of 1.26 Å, i.e. only slightly longer than free trans-HNNH (1.25 Å). The Fe2–N bond is 1.94 Å, which is longer than for all terminal NNH_2_ complexes (1.79–1.92 Å; the shortest bond if more than one). As can be seen in Fig. 4a, the HNNH group is stacked between His-195 and Ser-278, but it is not stabilised by any hydrogen bonds. It was found to be 9 kJ/mol more stable than the Fe6(HNNH_2_) complex (3 kJ/mol with the large basis set) and 40 kJ/mol more stable than the Fe3/7Fe4/5 complex (31 kJ/mol with the large basis set). We performed a full investigation of the BS states and the BS10-147 state turned out to be the lowest in energy, 32 kJ/mol more stable than the BS7-235 state. All states are shown in Table S2 and it can be seen that three additional states are within 3–8 kJ/mol, BS7-346, BS10-135 and BS6-157. The BS10-147 state had the lowest spin population on Fe2 (2.1), slightly less than for the other Fe ions, 2.4–3.2 (in absolute terms).

We found five additional complexes with trans-HNNH. The one binding to Fe6 was also quite low in energy, 10 kJ/mol less stable than the Fe2(trans) complex (19 kJ/mol with the large basis set), which is 1–16 kJ/mol higher than the Fe6(HNNH_2_) complex. As can be seen in Fig. 4b, HNNH forms a hydrogen bond to the alcohol O atom of homocitrate (1.86 Å), but this atom is also involved in an internal hydrogen bond with its own acetate group (1.50 Å) and the other proton of the substrate does not form any hydrogen bond. It turned out to be most stable in the BS10-135 state, but the BS10-147 was only 6 kJ/mol less stable. The spin population on Fe6, – 1.3, was much lower (in absolute terms) than for the other six Fe ions, 2.1–3.2.

The Fe4(trans) complex was also quite stable, 29 kJ/mol less stable than the Fe2(trans) structure (28 kJ/mol with the large basis set). It had a shorter Fe–N bond length of 1.88 Å, whereas the N–N bond is 1.26 Å. As can be seen from Fig. 4c, the HNNH group forms a weak hydrogen bond to S4A (2.31 Å). As expected, it has a low spin population on Fe4 (1.2, compared to 2.4–3.1 for the other Fe ions). The other trans-HNNH complexes were appreciably higher in energy, 69–158 kJ/mol above Fe2(trans).

**Fig. 5:**
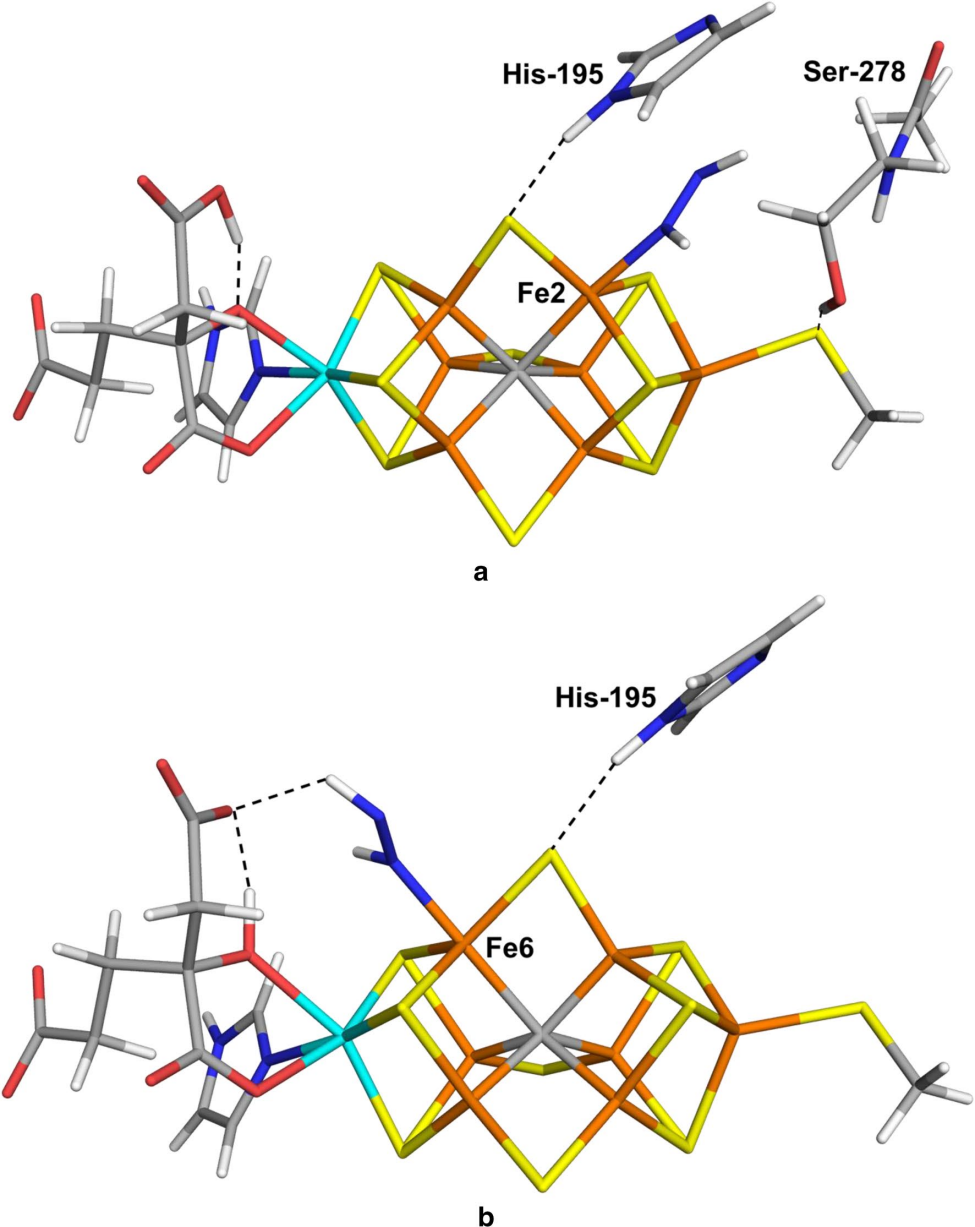
The most stable structures for end-on binding of cis-HNNH, obtained at the TPSS/def2-SV(P) level of theory, **a** Fe2(cis) and **b** Fe6(cis)

We studied also the corresponding cis-HNNH complexes. We found seven such complexes, as can be seen in Tables 2 and 3. Again, the complex involving binding to Fe2 [Fe2(cis)] turned out to be most stable, only 17 kJ/mol less stable than the Fe2(trans) complex (12 kJ/mol with the large basis set). The structure (Fig. 5a) and the spin population were very similar to the Fe2(trans) complex. HNNH is still stacked between His-195 and Ser-278, without forming any hydrogen bonds. We performed a full investigation of the BS states, and the best turned out to be BS10-147, 34 kJ/mol more stable than the BS7-235 state. Again, BS7-346, BS10-135 and BS6-157 were close in energy (1–6 kJ/mol).

The second-best structure was Fe6(cis), 48 kJ/mol less stable than Fe2(trans) (32 kJ/mol with the large basis set). It had Fe6–N and N–N bond lengths of 1.90 Å and 1.25 Å and the distal NH group forms a hydrogen bond to the acetate group of homocitrate (1.96 Å). The other structures turned out to be appreciably less stable, 67–187 kJ/mol less stable than Fe2(cis).

Considering the Fe Mulliken spin populations for all types of complexes (shown in Table S1), some general trends can be observed. First, the highest spin (throughout this paragraph, we discuss only the absolute values of the TPSS spin populations) is nearly always found on Fe1, 3.0–3.5. The only exceptions are when N_2_H_2_ binds to Fe1 and for the Fe2/4 and Fe3/4 complexes. The latter two complexes also differ that the largest spin population (on Fe3 or Fe2) is lower, 2.8–2.9, i.e. more similar to those of the second and third largest population for the other complexes. As mentioned above, the spin population is typically lowest on the Fe ions binding N_2_H_2_, but there are several exceptions, especially when N_2_H_2_ binds to several Fe ions. The lowest spin population varies from 0.03 for Fe4 in the Fe4Fe5(5) structure to 2.2 for Fe6 in the end-on Fe4 structure (in which Fe4 has a population of 2.3). The average is 1.2, illustrating that the spin is appreciably higher than for the N_2_ complexes studied by Dance, who got a spin population below 0.5 in 65% of the studied structures (and below 0.1 in 39% of the structures) [14]. There is no correlation between the lowest Fe spin population and the shortest Fe–N bond (*R* = –0.03). Fe6 and Fe7 often have relatively low spin populations (averages of 1.8 and 2.0 over all complexes), whereas Fe2–Fe5 have larger populations (averages of 2.5). Mo typically has a low spin population, 0–0.5, with an average of 0.2. However, in the end-on Fe7 complex, it is 0.9 and in the end-on Fe6Mo complex, it is 0.6. In about 75% of the complexes, the spin on Mo is negative.

### Calculations with a relaxed protein

In the calculations discussed so far, protein residues outside the QM system in Fig. 1 were kept fixed at their crystal positions. However, we observed a rather large variation in the MM energy of the surrounding protein (i.e. in the ${E}_{\text{MM123,}{\text{q}}_{1}= \text{0} }^{\text{CL}}-{E}_{\text{MM1,}{\text{q}}_{1}= \text{0} }^{\text{HL}}$ term in Eq. 2), up to 77 kJ/mol. This is much larger than in our previous studies (less than 10 kJ/mol) [22, 23], reflecting that the N_2_H_2_ ligand is rather large and may clash into the surrounding protein, so that its binding to the FeMo cluster may require significant reorganisation of the surrounding protein residues. Therefore, for all structures in Table 2, we also performed another QM/MM optimisation in which all MM residues with at least one atom within 6 Å of the QM system (always whole residues) were allowed to relax by a MM optimisation in each QM/MM geometry iteration.

The results of these calculations are also included in Tables 2and3. It can be seen that the relative energies change quite extensively (by up to 70 kJ/mol, both increasing or decreasing the relative stability), but the correlation between the two data sets is quite good, *R* = 0.89. In particular, Fe2(trans) remains the most stable complex and it is stabilised compared to the other low-energy complex, 23, 29, 86, 63, 85, 34 and 91 kJ/mol more stable than Fe6(HNNH_2_), Fe6(trans), Fe2(cis), Fe4(trans), Fe3/7Fe4/5, Fe6(cis) and Fe6 complexes. Thus, the Fe6(cis) complex becomes the fourth best structure. The Fe6(HCA) complex spontaneously reorganised to the Fe6(HNNH_2_) structure. In general, the Fe–N and N–N distances do not change much when the surroundings are allowed to relax as can be seen in Table 3. Fe5(cis) is the only structure that changes qualitatively, in that the HNNH ligand dissociates (it was bound weakly already in the structure with fixed surroundings, Fe5–N = 2.40 Å). The N–N distances change by no more than 0.03 Å. Likewise, the shortest Fe–N distance does not change by more than 0.05 Å.

### B3LYP results

All results described so far were obtained with the TPSS functional. However, for all structures, we also performed a B3LYP/def2-SV(P) geometry optimisation. The results of these are also collected in Tables 2 and 3. It can be seen that the two DFT functionals give quite similar structures for all complexes. In general, B3LYP gives a slightly shorter N–N bond, by 0.02 Å on average (0.01 Å for the isolated molecules). For the Fe–N distances, the variation is much larger and more varying (with differences from –0.5 to 0.3 Å), but on average, B3LYP gives slightly longer bonds (0.03 Å, median 0.02 Å). As for the relaxed structures, HNNH dissociated from Fe5(cis) with B3LYP, which is the only qualitative difference. However, in many cases, the relative lengths of Fe–N bonds to the same N atom change significantly.

The relative energies show larger and more systematic differences (the correlation coefficient to the TPSS energies is 0.84). B3LYP strongly disfavours all side-on structures, so that there are no such structures within 164 kJ/mol of the best structure. The end-on NNH_2_ structures (especially those involve more than one Fe ion) are also mostly disfavoured, but to a smaller extent. The Fe2(trans) complex is the most stable structure also with B3LYP. However, the second-best structure is Fe2(cis), which is only 18 kJ/mol less stable. The Fe6(HNNH_2_) and Fe6(trans) structures are still low in energy, 29 and 35 kJ/mol less stable than Fe2(trans). Next come the Fe6(cis), Fe6(HCA) and Fe4(trans) complexes, 64–75 kJ/mol less stable than Fe2(trans).

For the best structures, we also run B3LYP optimisations with the surrounding protein free to relax. These results are shown in the last column in Table 2. It can be seen that the Fe2(trans) structure is still best, being 34 kJ/mol more stable than the Fe4(trans) structure.

B3LYP and TPSS sometimes give differences in the most stable BS states. For example, the Fe2(trans) and Fe2(cis) structure are most stable in the BS8-236 state with B3LYP, but in the BS10-147 state with TPSS. Likewise, Fe6(trans) is most stable in the BS2-234 state with B3LYP, but BS10-135 with TPSS. This gives some significant differences in the geometry: for the Fe2(trans) complex, the Fe2–N bond length is 0.19 Å longer and the N–N bond is 0.03 Å shorter with B3LYP than with TPSS in the best BS states.

As usual, B3LYP gives larger and more similar Fe spin populations than TPSS (cf. Table S1). For the best Fe2(trans) structure, the spin on Fe2 with TPSS is 2.1, whereas the other Fe ions have spin populations of 2.4–3.2 (in absolute terms). However, with B3LYP, all Fe ions have 3.5–3.7 (3.6 on Fe2).

## Conclusions

We have studied the binding of the substrate to the FeMo cluster in nitrogenase. To reduce the very large number of possible structures, we have studied the binding of N_2_H_2_ to the E_0_ state of the cluster, rather than N_2_ to the E_4_ state. We have systematically studied side-on binding of HNNH and end-on binding of both NNH_2_ and HNNH to one, two or four Fe ions. We concentrated the study to the inner Fe ions (Fe2–Fe7), because both in this and previous studies [23], we have found that binding to the terminal Mo and Fe1 ions is unfavourable and Hoffman and coworkers have argued that N_2_ binding to Mo is unlikely [3, 72].

Interestingly, our results show that the binding of N_2_H_2_ is primarily determined by interactions with the surrounding protein and not by the intrinsic stability of the various binding modes. Thus, among the best structures, we find structures representing all four tested binding modes: side-on binding of HNNH, end-on binding of NNH_2_ and end-on binding of either cis- or trans-HNNH. This shows that it is absolutely necessary to study N_2_ binding with QM/MM methods; otherwise the results will be strongly biased by the selection of the QM model, unless all residues in the second coordination sphere of any atom in the FeMo cluster are included in the model. On the other hand, this also makes it harder to automatically set up all possible binding structures: in practice, we had to build up each binding mode by hand, carefully considering all surrounding residues. This should reduce the risk that some binding modes have been disfavoured by the use of a poor starting structure.

We find that the Fe2(trans) binding mode is most favourable, i.e. with trans-HNNH binding terminally to Fe2 (Fig. 4a). This mode is stabilised by HNNH being stacked between His-195 and Ser-278. The binding energy of HNNH to this structure (compared to the resting E_0_ state and trans-HNNH in a water-like continuum solvent) is favourable by 33 kJ/mol. The second most stable structure with TPSS has HNNH_2_ bound to Fe6. It is formed by a proton transfer from the homocitrate ligand and it is stabilised by two hydrogen bonds to the homocitrate ligand (Fig. 2c). The proton transfer stabilises the structure by 25–29 kJ/mol and the hydrogen bonds by 16–18 kJ/mol. This structure is 3–9 kJ/mol less stable than the Fe2(trans) structure at the TPSS level, but by 23 kJ/mol if the surroundings are allowed to relax. This structure is attractive, because it may explain why homocitrate is a compulsory ligand of the FeMo cluster and cannot be replaced by any related group, except erythro-1-fluorohomocitrate [73].

With B3LYP, instead the structure with cis-HNNH binding to Fe2 is second best, 18 or 46 kJ/mol less stable than Fe2(trans), depending on whether the surroundings are relaxed or not. This structure is fourth best with TPSS, 17 kJ/mol less stable than Fe2(trans). Two structures with trans-HNNH bound to Fe6 or Fe4 are also rather low in energy.

With TPSS, a structure with cis-HNNH bound side-on to the Fe3–Fe4–Fe5–Fe7 face of the FeMo cluster is also rather low in energy, 31–40 kJ/mol less stable than Fe2(trans). However, this structure is strongly disfavoured by B3LYP [183 kJ/mol less stable than Fe2(trans)] and also destabilised if the surroundings are relaxed [85 kJ/mol less stable than Fe2(trans) with TPSS].

Several other groups have studied the binding of N_2_ to the FeMo cluster. Dance studied the binding of N_2_ to the Fe2 or (mainly) Fe6 ions for a minimal cluster model with BLYP and the numerical DNP basis set [14, 74]. He compared 54 structures, differing in the number (0–4) and positions of protons bound to the cluster, the N_2_ binding mode (end-on or side-on) and direction, as well as the spin state, whereas only one BS state was considered, BS7-247. He found that N_2_ never bridges two metal ions, that end-on binding is more stable than side-on binding and that it is most favourable if N_2_ binds trans to the central carbide, which typically leads to cleavage of the Fe–C bond (but the carbide ion always retains at least five Fe–C bonds). The most stable structure had three protons (on S2B, Fe2 and Fe6) and N_2_ bound to Fe6, trans to C in the triplet state. However, in his suggested reaction mechanism, instead a structure with another proton on S3B and N_2_ binding side-on to Fe6 was selected, although it was 105 kJ/mol less stable. Moreover, as soon as it starts to be protonated, bridging structures were preferred. Therefore, for the HNNH state, he suggested a structure with HNNH bridging Fe2 and Fe6 asymmetrically (one N bridges Fe2 and Fe6, whereas the other binds only to Fe6. This is clearly not the most stable structure in our study, but it differs from our structures in that it contains three additional protons (still on S2B, Fe2 and Fe6). We do not observe any cleavage of the trans Fe–C bonds in our N_2_H_2_-bond structures, only a slight elongation (0.05 Å for the best Fe2(trans) structure, 0.13 Å for the Fe6(HNNH_2_) and Fe6(HCA) structures, but a shortening by ~ 0.02 Å for the Fe4(cis) and Fe3/7Fe4/5 structures, compared to the crystal structure of the resting state [6]). This is probably an effect of the inclusion of the surrounding protein with its steric and electrostatic stabilisation.

Hallmen and Kästner also studied the binding of N_2_ to the FeMo cluster with the PBE functional and plane-wave basis set for a minimal cluster model of the E_2_ state [33]. They considered 12 structures, differing in the binding position of N_2_ (Fe2, Fe3, Fe6, Fe7 or Mo), whereas the two protons were kept on S2B and S5A. In contrast to Dance, they reported several bridging structures, but the structure with N_2_ binding end-on to Fe7 (not trans to C) and with a cleaved Fe7–S5A bond was best, 10 kJ/mol more stable than a structure with N_2_ binding end-on to Mo. In an earlier study of the full reaction mechanism (with a central N^3–^ ion), Kästner and Blöchl suggested that HNNH bridges Fe3 and Fe7 first symmetrically and then asymmetrically [32]. Neither of these structures are supported by our calculations, showing that systematic searches of all binding possibilities are needed, as well as a detailed account of the surrounding protein with its sterical and hydrogen-bonding interactions.

The electronic structure of the FeMo cluster is an important ingredient of DFT studies of nitrogenase. Most groups argue that it does not significantly affect the results and that it is enough to restrict the study to a few BS states [14, 16]. Unfortunately, the results in Table S2 indicate that this an oversimplification. The most stable BS can be either BS10-147, BS10-135, BS7-235, BS2-234 or (only B3LYP) BS8-236. Thus, these belong to four out of Noodleman’s ten types of BS states, without any clear logical connection. Moreover, in total, 18 BS states (i.e. more than half of the 35 possible BS states) are within 10 kJ/mol of the best state for at least one of the 18 investigated structures (including all Noodleman states, except BS1 and BS9) and all 35 BS states are within 50 kJ/mol of the best state for at least one structure. This makes it hard to predict beforehand which state will be most favourable. It also indicates that the automatic procedure employed by Kästner and Blöchl [32], indicating that the best states always belong to BS6 or BS7, does not work properly. Even worse, if we select to always use the BS10-147 state, which is best for six structures and within 10 kJ/mol of the best BS state for four additional structures, it is up to 120 kJ/mol worse than the best BS state for other structures, which could lead to erroneous predictions. BS7-235, which is best for six structures and within 10 kJ/mol for additional two, seems to be a slightly better choice, but it can still give errors of up to 89 kJ/mol (or BS2-234, which may give errors of 82 kJ/mol). Therefore, we recommend a complete BS investigation for the best structures, including full geometry optimisations.

Finally, we note that it is satisfying that our all six best structures involve N_2_H_2_ binding to Fe2 and Fe6, because experimental observations have suggested the Fe2–Fe3–Fe6–Fe7 face as the reactive side of the cluster [3, 72, 75]. This shows that our QM/MM approach works properly and is accurate enough to find the most reactive sites without restricting the search by experimental information. This makes our structures the most likely candidates for the N_2_-bound structure of nitrogenase. In future investigations, we will study what implications this finding has for the interpretation of the E_4_ state and how the N_2_-bound states may continue to react and form the NH_3_ product. We will also investigate the possible dissociation of S2B [7, 76], which bridges the same Fe2 and Fe6 ions, forming a natural binding site between these two ions. In several of our studied structures, this ligand dissociates from one of the two Fe ions when the substrate binds (but never from both).

## Electronic supplementary material

Below is the link to the electronic supplementary material.

Supplementary file1 (PDF 772 kb)
