# Supplementary material for: N2H2 binding to the nitrogenase FeMo cluster studied by QM/MM methods
Source: J Biol Inorg Chem. 2020 Apr 7;25(3):521–40. doi: 10.1007/s00775-020-01780-5 (PMC7186253; doi:10.1007/s00775-020-01780-5)
Supplement: Supplementary file 1 — Supplementary file1 (PDF 772 kb) [file 775_2020_1780_MOESM1_ESM.pdf]

N_2H_2 binding to the nitrogenase FeMo cluster studied by QM/MM methods

Lili Cao and Ulf Ryde *

Department of Theoretical Chemistry, Lund University, Chemical Centre, P. O. Box 124,

SE-221 00 Lund, Sweden

Correspondence to Ulf Ryde, E-mail: Ulf.Ryde@teokem.lu.se,

Tel: +46 – 46 2224502, Fax: +46 – 46 2228648

2020-04-01

Table S1. Mulliken spin populations for the Fe and Mo ions in the FeMo cluster obtained from TPSS and B3LYP calculations with the def2-SV(P) basis set for the various states.

Structure	BS	TPSS								B3LYP							
		Fe1	Fe2	Fe3	Fe4	Fe5	Fe6	Fe7	Mo	Fe1	Fe2	Fe3	Fe4	Fe5	Fe6	Fe7	Mo
**End-on NNH_2 binding**																	
Fe1	BS7-235	1.7	-2.4	-3.2	3.0	-2.9	2.4	3.0	-0.4	2.4	-3.5	-3.7	3.7	-3.5	3.5	3.6	-1.1
Fe2	BS7-235	3.3	-2.5	-2.9	2.9	-2.6	0.4	2.2	0.1	3.7	-3.8	-3.7	3.6	-3.5	3.2	3.3	-1.0
Fe3	BS10-147	3.2	-2.6	-2.0	3.1	-2.4	-1.3	2.3	0.2	3.6	-3.4	-2.5	3.7	-3.3	-3.1	3.5	0.9
Fe4	BS7-235	3.2	-2.7	-2.8	2.3	-2.9	2.2	2.4	-0.1	3.6	-3.7	-3.6	3.1	-3.6	3.3	3.4	-0.8
Fe5	BS7-235	3.4	-3.4	-3.1	2.5	-2.5	2.3	2.1	-0.2	3.6	-3.8	-3.8	3.4	-3.5	3.4	3.4	-1.1
Fe6	BS10-147	-3.2	2.6	2.7	-3.0	2.7	1.6	-2.2	-0.3	-3.7	3.6	3.6	-3.6	3.6	2.1	-3.5	-0.7
Fe6(HCA)	BS10-135	-3.2	2.8	-3.0	2.8	-2.5	1.6	2.7	-0.2	-3.7	3.6	-3.7	3.5	-3.5	2.2	3.5	-0.9
Fe6($HNNH_2$)	BS10-135	-3.2	2.9	2.8	-3.0	2.8	1.8	-2.5	-0.3	-3.7	3.6	3.6	-3.6	3.6	2.7	-3.5	-0.8
Fe6(S2B)	BS10-127	-3.3	-2.8	2.9	2.8	2.2	1.5	-2.5	-0.1	-3.7	-3.5	3.5	3.4	3.0	2.6	-3.5	-0.7
Fe7	BS7-235	3.4	-2.8	-3.1	3.1	-2.6	2.7	1.0	-0.9	3.7	-3.6	-3.7	3.7	-3.5	3.6	2.1	-1.3
Fe3(S2A)	BS7-235	3.1	-3.0	-2.9	2.5	-2.8	2.2	2.1	-0.1	3.6	-3.7	-3.7	3.5	-3.6	3.3	3.3	-0.8
Fe2/4	BS7-235	2.5	-2.5	-2.8	1.8	-2.6	2.3	2.5	-0.2	3.5	-3.6	-3.6	3.4	-3.5	3.3	3.4	-1.0
Fe3/4	BS7-235	2.6	-2.9	-2.0	1.7	-2.7	2.4	2.5	-0.2	3.6	-3.7	-3.7	3.6	-3.5	3.4	3.4	-1.0
Fe2/6/7	BS7-235	3.3	-2.0	-2.8	2.8	-2.8	1.8	0.7	-0.2	3.7	-3.2	-3.7	3.5	-3.5	3.2	2.5	-1.1
Fe2/6(5)	BS10-147	3.3	-2.1	-2.7	3.1	-2.7	-0.8	2.3	0.3	3.7	-2.8	-3.5	3.6	-3.4	-2.7	3.5	1.2
Fe3/7(2)	BS5-256	3.4	-2.6	0.1	3.0	-2.7	-2.1	1.3	0.4	3.7	-3.8	2.4	3.5	-3.5	-3.5	2.1	1.1
Fe3/7(3)	BS7-235	3.3	-2.9	-2.1	3.0	-2.7	2.1	0.5	0.1	3.7	-3.7	-3.6	3.6	-3.5	3.2	2.3	-0.7
Fe4/5(2)	BS7-235	3.3	-2.8	-2.9	1.7	-2.6	2.2	2.3	-0.3	3.6	-3.7	-3.6	3.1	-3.4	3.4	3.4	-1.2
Fe4/5(5)	BS7-235	3.3	-2.7	-2.8	1.5	-2.8	2.3	2.5	-0.2	3.8	-3.6	-3.6	2.4	-3.6	3.4	3.5	-1.0
Fe2/3/6/7	BS7-235	3.3	-1.3	-2.9	2.9	-2.7	2.3	-1.1	0.1	3.7	-2.7	-3.6	3.6	-3.3	3.6	-2.3	0.6
Fe2/4/5/6	BS7-235	3.3	-3.0	-3.0	1.7	-2.1	2.4	2.3	-0.3	3.6	-3.7	-3.7	2.9	-3.2	3.4	3.4	-1.1
Fe3/4/5/7	BS7-235	3.3	-2.7	-2.8	1.6	-2.5	2.5	1.2	-0.1	3.8	-3.6	-3.6	2.4	-3.5	3.4	2.4	-0.7
**Side-on cis-HNNH binding**																	
Fe2Fe2	BS10-147	3.3	-2.3	-2.7	3.1	-2.3	-1.2	2.6	0.1	3.7	-2.6	-3.4	3.7	-3.3	-3.0	3.5	0.8
Fe4Fe4	BS10-147	-3.2	2.3	2.7	-2.6	1.8	2.1	-2.6	-0.1	-3.7	3.4	3.4	-3.4	2.8	2.8	-3.5	-0.6
Fe5Fe5	BS10-146	3.2	-2.7	-2.5	3.1	-1.6	2.7	-1.8	0.1	3.7	-3.5	-3.3	3.7	-2.5	3.5	-3.0	0.9
Fe6Fe6	BS7-235	3.4	-3.1	-2.9	3.1	-2.7	1.2	1.8	0.2	3.7	-3.6	-3.8	3.6	-3.5	3.0	3.3	-0.8
Fe7Fe7	BS5-256	3.2	-2.8	1.0	3.0	-2.7	-1.9	1.2	0.3	3.6	-3.7	2.4	3.5	-3.6	-3.5	2.7	0.7
Fe2/3Fe6/7	BS7-235	3.3	-2.3	-2.0	2.9	-2.7	0.9	0.8	-0.4	3.7	-3.8	-3.8	3.5	-3.5	2.4	3.5	-1.2

Fe2/6Fe3/7	BS7-235	3.0	-2.4	-2.1	0.9	-2.5	2.2	1.9	-0.2	3.7	-3.6	-3.6	3.5	-3.5	2.8	2.7	-0.9
Fe2/6Fe3/7tr[a]	BS6-156	3.1	-1.6	-1.1	-2.9	2.7	1.1	0.0	-0.4	-3.6	3.0	2.6	3.6	-3.5	-3.5	2.4	0.9
Fe2/4Fe5/6	BS2-234	3.1	-1.6	-2.7	-1.4	0.2	1.4	2.5	-0.5	3.8	-2.6	-3.5	-3.7	2.8	2.6	3.4	-2.1
Fe2/6Fe4/5	BS3-134	3.1	-1.9	1.7	1.9	-1.3	-0.5	-2.4	0.3	3.7	-3.0	3.5	2.7	-2.7	-2.7	-3.5	2.2
Fe2/6Fe4	BS7-235	3.2	-1.6	-2.8	1.4	-2.7	1.2	2.4	-0.2	3.6	-2.7	-3.7	2.8	-3.5	2.7	3.4	-1.2
Fe3/7Fe4/5	BS2-234	3.0	-2.7	-1.6	-1.8	1.1	2.4	0.8	-0.5	3.8	-3.5	-3.5	-3.5	2.7	3.5	2.7	-2.3
Fe2Fe7	BS7-235	3.3	-2.6	-3.0	3.0	-2.8	2.2	0.9	-0.2	3.7	-3.6	-3.8	3.5	-3.5	3.4	2.2	-0.8
Fe3Fe6	BS10-147	3.5	-3.3	-1.3	2.8	-2.7	-1.1	2.4	0.2	3.7	-3.7	-2.0	3.6	-3.5	-2.4	3.5	0.8
Fe2Fe6(3)	BS8-245	3.3	-2.0	-2.6	3.0	-2.7	-0.7	2.2	0.3	3.7	-2.9	-3.5	3.7	-3.4	-2.4	3.5	1.0
Fe2Fe6(5)	BS7-235	3.2	-2.8	-3.0	2.9	-2.7	1.1	2.3	-0.2	3.7	-3.6	-3.8	3.5	-3.5	2.3	3.4	-1.1
Fe3/7Fe3(2)	BS8-245	-3.5	2.9	-0.5	3.0	2.6	-2.4	-1.1	0.2	-3.8	3.6	-1.3	3.6	3.5	-3.4	-2.7	0.9
Fe3Fe7(3)	BS5-256	3.3	-2.6	1.5	3.0	-2.7	-1.8	0.3	0.5	3.7	-3.7	2.5	3.6	-3.5	-3.4	2.1	1.4
Fe45Fe5(2)	BS7-235	3.3	-2.7	-2.8	0.4	-2.0	2.3	2.5	-0.4	3.8	-3.6	-3.6	1.7	-3.4	3.4	3.4	-1.1
Fe4Fe5(5)	BS6-167	3.4	-2.8	-3.2	0.0	-1.2	2.3	2.3	-0.3	3.8	-3.5	-3.6	-1.1	-2.0	3.4	3.4	-1.3
**End-on trans-HNNH binding**																	
Fe2(trans)	BS10-147	-3.2	2.1	3.0	-3.0	2.6	2.4	-2.4	-0.3	3.7	-3.6	-3.7	3.6	3.5	-3.7	3.6	-2.4
Fe3(trans)	BS10-146	-3.3	2.9	2.0	-2.9	2.6	-2.4	2.6	-0.2	-3.7	3.7	2.3	-3.6	3.4	-3.5	3.4	-0.7
Fe4(trans)	BS7-235	3.3	-2.7	-2.8	1.2	-2.8	2.3	2.5	-0.2	3.8	-3.6	-3.6	2.0	-3.6	3.4	3.4	-1.0
Fe5(trans)	BS10-147	3.2	-2.2	-2.7	3.0	-1.5	-2.0	2.8	0.1	3.7	-3.3	-3.5	3.7	-2.1	-3.1	3.5	1.1
Fe6(trans)	BS10-147	3.2	-2.1	-2.6	3.0	-2.3	-1.3	2.7	0.1	-3.5	3.8	3.8	3.8	-3.4	-3.5	-3.4	2.3
Fe7(trans)	BS10-147	3.1	-2.1	-2.2	3.1	-2.6	-1.1	2.6	0.0	3.7	-2.7	-3.0	3.6	-3.4	-3.0	3.6	1.0
**End-on cis-HNNH binding**																	
MoFe6	BS7-235	3.0	-2.5	-2.6	2.8	-2.9	2.0	2.5	-0.6	3.5	-3.4	-3.4	3.5	-3.5	3.2	3.4	1.7
Fe2(cis)	BS10-147	-3.2	2.1	3.0	-2.9	2.5	2.4	-2.5	-0.3	3.7	-3.6	-3.7	3.6	3.5	-3.7	3.6	-2.4
Fe3(cis)	BS10-146	-3.3	2.9	2.0	-2.8	2.6	-2.5	2.6	-0.2	-3.7	3.7	2.4	-3.6	3.4	-3.5	3.4	-0.7
Fe4(cis)	BS7-235	3.3	-2.7	-2.9	1.4	-2.8	2.3	2.5	-0.2	3.8	-3.6	-3.6	2.1	-3.6	3.4	3.4	-1.0
Fe5(cis)	BS7-235	3.2	-3.0	-3.0	2.5	-2.8	1.8	2.3	0.0	3.6	-3.8	-3.8	3.4	-3.6	3.3	3.4	-1.0
Fe6(cis)	BS10-135	-3.2	2.7	-2.9	2.8	-2.5	1.4	2.7	-0.1	-3.7	3.6	-3.7	3.5	-3.5	2.1	3.5	-0.8
Fe7(cis)	BS7-235	3.2	-2.8	-2.4	2.9	-2.8	2.0	0.9	0.0	3.7	-3.7	-3.5	3.6	-3.5	3.3	2.2	-0.7

[a] In this structure, one of the protons of HNNH has moved to S5A, whereas the ligand has taken a proton from Arg-96 (cf. Figure S2).

Table S2. Relative energies of the 35 BS states for some of the studied structures, obtained with TPSS (TP) or B3LYP(B3).

BS	Fe2(trans)		Fe6-HNNH2		Fe6(trans)		Fe2(cis)		Fe6		Fe6(HCA)		Fe3/4	Fe4/5(5)		Fe3(S2A)	Fe3/7Fe4/5		Fe2Fe6(3)	Fe2Fe6(5)
state	TP	B3	TP	B3	TP	B3	TP	B3	TP	B3	TP	B3	TP	TP	B3	B3	TP	B3	TP	TP
BS1-567	86	186	101	161		220	97	179		254	44	35	83	64	218	207			56	
BS2-234	19	2	43	22	35	0	18	3	41	50	32	50	48	4	83	18	0	70	60	26
BS3-123	55	8	5	112	37	32	55	7	47	113	58	59	72	67	119	31	57	104	56	71
BS3-124	44	14	53	67		20	55	11	66	70	44	123	66	40	101	55	61	114	48	65
BS3-134	64	32	48	30	43	11	68	44	71	78	44	36	73	28	88	73	58	69	68	39
BS4-257	79	149	55	86	58	91	80	135	71	124	53	112	25	24	76	134	44	69	67	39
BS4-356	30	64	44	74	33	68	27	69		131	54	138	55	20	80	123	45	77	51	0
BS4-467	41	95	63	95		158	38	90	78	198			64	48	216	161	38	107	0	90
BS5-256		120	44	105	58	108		137		153			54	64	140	162			69	0
BS5-267		145	68	94		167		145	90	202			50	22	132	193			53	115
BS5-357	45	89	49	74	55	127	51	86	81	155	57	122	61	47	117	148			51	60
BS5-367	53	86	77	97	32	194	46	87	90	187	80	194	54	32	113	186	53	196	37	60
BS5-456	51	93	76	110		89	50	120	77	224	71	187	63	78	200	130			56	83
BS5-457	59	94	53	97	45	96	59	96		229	56	142	84	8	201	117	77	123	56	54
BS6-156	18	72	19	62	29	69	17	69	39	118	35	159	19		110	140	20	46	16	66
BS6-157	8	62	21	33	14	75	6	55	34	150	16	91	45	4	107	138	33	147	16	37
BS6-167	22	87	36	117	34	123	17	85	69	118			53	26	105	132	18	36	0	0
BS7-235	32	24	17	10		48	34	89	51	0	8	19	0	0	0	0	19	0	3	0
BS7-247	45	27	8	8	45	56	40	29	51	92	38	124	33	8	122	47	38	62	77	49
BS7-346	3	15	5	36		41	1	14	36	85	65	87	75	50	125	53	38	67	0	67
BS8-236	28	0	11	51		51	26	0	86	58		223	52	23	83	72	67	73	40	74
BS8-237	42	79	40	24	49	64	46	97	66	45	42	85	13	9	49	66			40	40
BS8-245	49	74	45	33	55	67	48	128	87	108	54	109	49	65	94	70	38	56	62	75
BS8-246	31	15	13	87		49	32	9	122	164	64	171	53	68	139	90			52	87
BS8-345	18	18	19	9	45	18	17	16	51	113	46	79	76	61	122	70	80	51	31	43
BS8-347	17	16	14	4	41	64	16	13	41	141	46	86	76	79	156	74			33	37
BS9-126	26	28	47	86	53	87	25	26	75	109	45	140	86	60	102	107	95	143	29	90
BS9-137	33	44	38	28	45	85	34	41	73	166	42	97	71	45	79	105	25		65	68
BS9-145	29	25	38	17	37	49	26	23	72	118	37	20	43	31	96	95			54	58
BS10-125	18	18	28	6	13	29	14	17	42	59	14	12	49	22	65	41	69	50	21	38
BS10-127	15	40	24	7	15	44	16	42	32	82	14	55	53	35	75	63	66	55	25	41
BS10-135	5	20	5	4	0	32	5	15	43	79	0	0	46	12	59	60	27	76	37	64
BS10-136	33	26	29	54	56	47	32	29	46	85	67	140	38	20	38	86	48	29	74	50
BS10-146	26	20	26	46	51	60	37	18	41	99	20	84	45	3	115	91	44	44	4	37
BS10-147	0	9	0	0	6	54	0	10	0	120	8	58	22	22	112	70	24	86	0	21

Figure S1. The QM/MM geometry of the Fe3/4 structure.

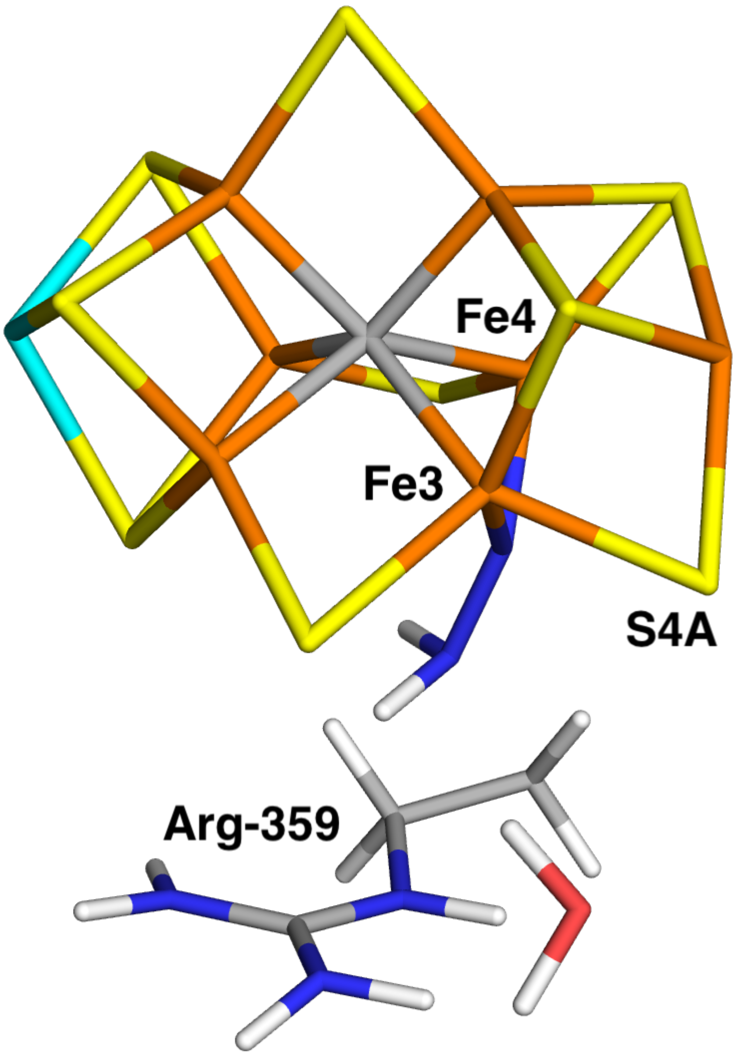

Figure S2. The QM/MM geometry of the Fe2/6Fe3/7tr structure.

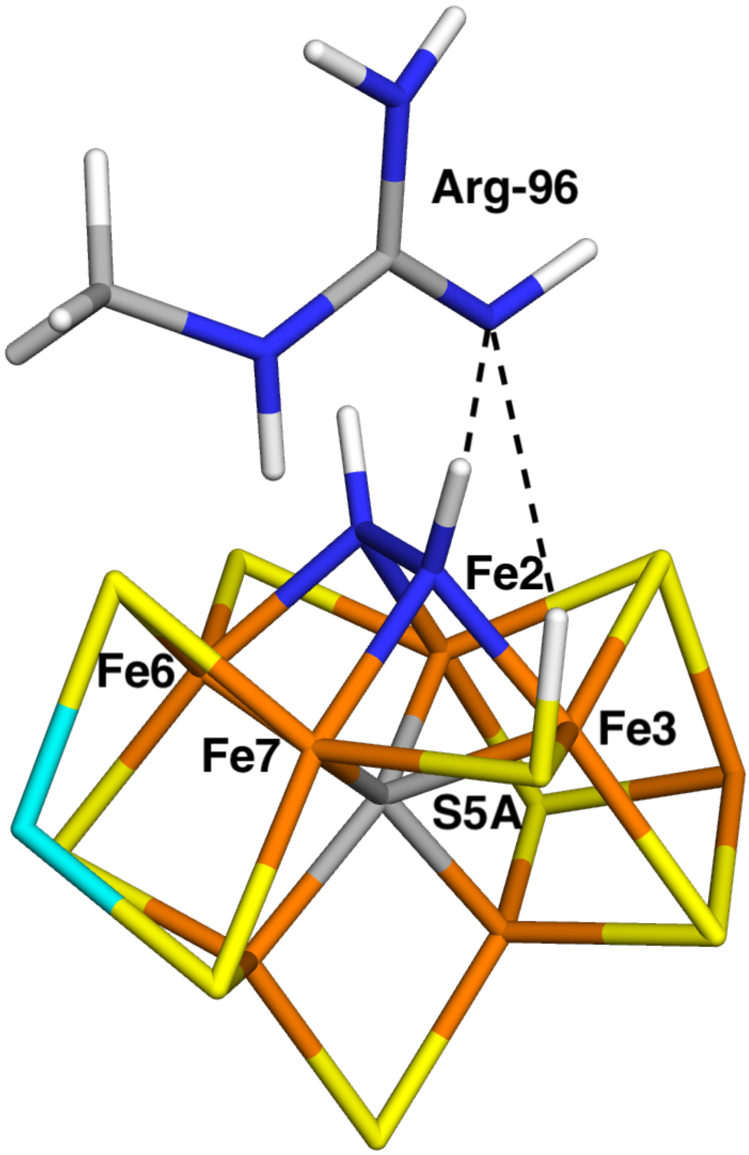

Table S3. Coordinates of the best structures in PDB format.

```
Fe6(HCA)
REMARK Energies (QM/MM, QM+ptch) =  -17129.752558 -16290.129409     -5.284382 H
ATOM      1  H   ???     1       3.500   2.240 -29.687
ATOM      2  C   ???     1       3.906   2.095 -30.706
ATOM      3  H   ???     1       5.011   2.115 -30.641
ATOM      4  H   ???     1       3.594   1.112 -31.093
ATOM      5  N   ???     1       3.476   3.148 -31.617
ATOM      6  H   ???     1       3.968   4.049 -31.522
ATOM      7  C   ???     1       2.847   2.926 -32.785
ATOM      8  N   ???     1       1.989   1.881 -32.911
ATOM      9  H   ???     1       1.813   1.274 -32.108
ATOM     10  H   ???     1       1.787   1.493 -33.843
ATOM     11  N   ???     1       3.066   3.743 -33.817
ATOM     12  H   ???     1       3.624   4.614 -33.675
ATOM     13  H   ???     1       2.416   3.767 -34.609
ATOM     14  H   ???     1       7.276   1.562 -41.618
ATOM     15  C   ???     1       7.581   2.030 -40.684
ATOM     16  N   ???     1       8.438   3.113 -40.698
ATOM     17  C   ???     1       8.511   3.514 -39.434
ATOM     18  H   ???     1       9.078   4.358 -39.042
ATOM     19  N   ???     1       7.746   2.736 -38.622
ATOM     20  H   ???     1       7.595   2.940 -37.616
ATOM     21  C   ???     1       7.128   1.786 -39.401
ATOM     22  H   ???     1       6.433   1.057 -38.985
ATOM     23  H   ???     1       2.978   9.557 -40.118
ATOM     24  C   ???     1       3.104   8.705 -39.422
ATOM     25  H   ???     1       2.509   7.840 -39.762
ATOM     26  H   ???     1       2.796   9.023 -38.413
ATOM     27  S   ???     1       4.918   8.301 -39.430
ATOM     28  H   ???     1       6.637   8.643 -42.923
ATOM     29  C   ???     1       7.435   7.842 -42.957
ATOM     30  H   ???     1       8.006   8.022 -43.881
ATOM     31  H   ???     1       8.092   7.980 -42.081
ATOM     32  C   ???     1       6.837   6.419 -43.009
ATOM     33  O   ???     1       7.262   5.638 -43.871
ATOM     34  N   ???     1       5.923   6.124 -42.064
ATOM     35  H   ???     1       5.588   6.846 -41.409
ATOM     36  C   ???     1       5.391   4.763 -41.928
ATOM     37  H   ???     1       6.246   4.093 -42.128
ATOM     38  C   ???     1       4.922   4.521 -40.489
ATOM     39  H   ???     1       4.592   3.469 -40.417
ATOM     40  H   ???     1       5.757   4.667 -39.771
ATOM     41  O   ???     1       3.807   5.335 -40.100
ATOM     42  H   ???     1       4.172   6.176 -39.724
ATOM     43  H   ???     1       4.607   4.494 -42.656
ATOM     44  H   ???     1      13.632  11.342 -31.927
ATOM     45  C   ???     1      12.719  10.654 -31.970
ATOM     46  H   ???     1      12.873   9.949 -31.129
ATOM     47  H   ???     1      11.736  11.192 -31.788
ATOM     48  C   ???     1      12.887   9.818 -33.252
ATOM     49  O   ???     1      14.017   9.620 -33.703
ATOM     50  N   ???     1      11.789   9.210 -33.775
ATOM     51  H   ???     1      10.829   9.423 -33.469
ATOM     52  C   ???     1      11.956   8.128 -34.737
ATOM     53  H   ???     1      12.913   7.632 -34.528
ATOM     54  H   ???     1      11.128   7.417 -34.566
ATOM     55  C   ???     1      11.982   8.526 -36.221
ATOM     56  O   ???     1      12.994   8.407 -36.914
ATOM     57  N   ???     1      10.779   8.969 -36.715
ATOM     58  H   ???     1       9.939   8.819 -36.131
ATOM     59  C   ???     1      10.594   9.155 -38.142
ATOM     60  H   ???     1      11.591   9.303 -38.593
ATOM     61  H   ???     1      10.153   8.254 -38.606
ATOM     62  C   ???     1       9.676  10.291 -38.578
ATOM     63  O   ???     1       9.529  10.513 -39.786
ATOM     64  N   ???     1       9.053  11.005 -37.603
ATOM     65  H   ???     1       9.117  10.677 -36.631
ATOM     66  C   ???     1       8.100  12.061 -37.928
ATOM     67  H   ???     1       8.334  12.410 -38.945
ATOM     68  H   ???     1       7.043  11.659 -37.929
ATOM     69  C   ???     1       8.207  13.251 -36.958
ATOM     70  O   ???     1       8.476  14.390 -37.364
ATOM     71  N   ???     1       7.918  12.931 -35.677
ATOM     72  H   ???     1       7.748  11.944 -35.440
ATOM     73  C   ???     1       7.844  13.898 -34.572
ATOM     74  H   ???     1       6.991  14.578 -34.788
ATOM     75  C   ???     1       7.531  13.116 -33.288
ATOM     76  H   ???     1       8.318  12.354 -33.114
ATOM     77  H   ???     1       7.559  13.819 -32.433
ATOM     78  C   ???     1       6.159  12.415 -33.323
ATOM     79  H   ???     1       5.357  13.183 -33.320
ATOM     80  H   ???     1       6.037  11.822 -34.252
ATOM     81  C   ???     1       5.963  11.462 -32.142
ATOM     82  H   ???     1       6.654  10.600 -32.234
ATOM     83  H   ???     1       6.189  11.992 -31.195
ATOM     84  N   ???     1       4.572  10.996 -32.105
ATOM     85  H   ???     1       3.963  11.178 -32.917
ATOM     86  C   ???     1       3.988  10.366 -31.079
ATOM     87  N   ???     1       4.663  10.163 -29.937
ATOM     88  H   ???     1       5.611  10.533 -29.826
ATOM     89  H   ???     1       4.323   9.520 -29.215
ATOM     90  N   ???     1       2.709   9.938 -31.210
ATOM     91  H   ???     1       2.243  10.183 -32.094
ATOM     92  H   ???     1       2.097   9.951 -30.391
ATOM     93  H   ???     1       8.731  14.564 -34.480
ATOM     94  H   ???     1       8.402   7.469 -28.176
ATOM     95  C   ???     1       9.346   7.522 -28.725
ATOM     96  N   ???     1       9.536   6.791 -29.884
ATOM     97  C   ???     1      10.842   6.862 -30.166
ATOM     98  H   ???     1      11.343   6.389 -31.011
ATOM     99  N   ???     1      11.483   7.626 -29.249
```

```
ATOM    100  H   ???     1      12.495   7.849 -29.266
ATOM    101  C   ???     1      10.555   8.051 -28.319
ATOM    102  H   ???     1      10.834   8.719 -27.505
ATOM    103  O   ???     1      10.648   5.253 -25.654
ATOM    104  C   ???     1      10.944   4.627 -26.735
ATOM    105  O   ???     1      12.096   4.379 -27.167
ATOM    106  C   ???     1       9.736   4.221 -27.610
ATOM    107  H   ???     1       8.870   4.020 -26.953
ATOM    108  H   ???     1       9.484   5.132 -28.179
ATOM    109  C   ???     1       9.914   3.093 -28.634
ATOM    110  H   ???     1      10.887   3.196 -29.142
ATOM    111  H   ???     1       9.916   2.101 -28.137
ATOM    112  C   ???     1       8.824   3.120 -29.749
ATOM    113  O   ???     1       9.238   4.082 -30.746
ATOM    114  H   ???     1       9.291   3.680 -31.766
ATOM    115  C   ???     1       7.507   3.632 -29.121
ATOM    116  O   ???     1       7.111   4.794 -29.520
ATOM    117  O   ???     1       6.903   2.972 -28.247
ATOM    118  C   ???     1       8.570   1.740 -30.392
ATOM    119  H   ???     1       8.109   1.080 -29.636
ATOM    120  H   ???     1       7.818   1.883 -31.199
ATOM    121  C   ???     1       9.800   1.030 -31.019
ATOM    122  O   ???     1      10.562   1.759 -31.739
ATOM    123  O   ???     1       9.918  -0.201 -30.831
ATOM    124 FE   ???     1       7.919   7.752 -33.107
ATOM    125 MO   ???     1       7.993   5.792 -31.254
ATOM    126 FE   ???     1       6.995   8.481 -35.338
ATOM    127 FE   ???     1       5.813   6.429 -32.629
ATOM    128 FE   ???     1       7.932   5.099 -33.834
ATOM    129 FE   ???     1       6.847   5.999 -36.046
ATOM    130 FE   ???     1       5.514   7.776 -37.309
ATOM    131 FE   ???     1       4.871   7.134 -34.849
ATOM    132  C   ???     1       6.738   6.827 -34.294
ATOM    133  S   ???     1       7.736   7.663 -37.360
ATOM    134  S   ???     1       9.609   6.325 -32.920
ATOM    135  S   ???     1       4.739   5.702 -36.646
ATOM    136  S   ???     1       7.977   4.166 -35.895
ATOM    137  S   ???     1       8.510   9.631 -34.196
ATOM    138  S   ???     1       6.429   4.298 -32.308
ATOM    139  S   ???     1       4.914   9.276 -35.636
ATOM    140  S   ???     1       6.748   7.812 -31.168
ATOM    141  S   ???     1       3.666   6.744 -32.982
ATOM    142  N   ???     1       8.936   3.667 -33.235
ATOM    143  H   ???     1       9.852   1.950 -33.247
ATOM    144  N   ???     1       9.292   2.623 -33.836
ATOM    145  H   ???     1       9.065   2.536 -34.853
ATOM    146  O   ???     1       4.507   5.540 -29.443
ATOM    147  H   ???     1       5.462   5.257 -29.486
ATOM    148  H   ???     1       4.439   6.273 -30.095
ATOM    149  O   ???     1       1.061   8.394 -34.250
ATOM    150  H   ???     1       1.915   8.051 -33.887
ATOM    151  H   ???     1       0.548   8.690 -33.441
END

Fe6
REMARK Energies (QM/MM, QM+ptch) =  -17129.745891 -16290.117291
ATOM      1  H   ???     1       3.496   2.241 -29.688
ATOM      2  C   ???     1       3.896   2.098 -30.711
ATOM      3  H   ???     1       5.002   2.103 -30.655
ATOM      4  H   ???     1       3.569   1.120 -31.101
ATOM      5  N   ???     1       3.469   3.163 -31.609
ATOM      6  H   ???     1       3.961   4.062 -31.495
ATOM      7  C   ???     1       2.856   2.958 -32.789
ATOM      8  N   ???     1       2.001   1.913 -32.938
ATOM      9  H   ???     1       1.825   1.293 -32.145
ATOM     10  H   ???     1       1.805   1.535 -33.876
ATOM     11  N   ???     1       3.086   3.793 -33.804
ATOM     12  H   ???     1       3.634   4.667 -33.635
ATOM     13  H   ???     1       2.449   3.826 -34.606
ATOM     14  H   ???     1       7.279   1.558 -41.619
ATOM     15  C   ???     1       7.594   2.016 -40.683
ATOM     16  N   ???     1       8.457   3.094 -40.689
ATOM     17  C   ???     1       8.574   3.450 -39.415
ATOM     18  H   ???     1       9.155   4.281 -39.014
ATOM     19  N   ???     1       7.834   2.644 -38.605
ATOM     20  H   ???     1       7.699   2.821 -37.592
ATOM     21  C   ???     1       7.181   1.730 -39.397
ATOM     22  H   ???     1       6.491   0.995 -38.983
ATOM     23  H   ???     1       2.981   9.557 -40.119
ATOM     24  C   ???     1       3.115   8.706 -39.423
ATOM     25  H   ???     1       2.538   7.831 -39.770
ATOM     26  H   ???     1       2.783   9.021 -38.420
ATOM     27  S   ???     1       4.941   8.335 -39.405
ATOM     28  H   ???     1       6.638   8.643 -42.923
ATOM     29  C   ???     1       7.436   7.841 -42.958
ATOM     30  H   ???     1       8.006   8.022 -43.881
ATOM     31  H   ???     1       8.092   7.980 -42.081
ATOM     32  C   ???     1       6.837   6.419 -43.009
ATOM     33  O   ???     1       7.258   5.639 -43.874
ATOM     34  N   ???     1       5.924   6.124 -42.063
ATOM     35  H   ???     1       5.599   6.846 -41.401
ATOM     36  C   ???     1       5.390   4.764 -41.924
ATOM     37  H   ???     1       6.245   4.093 -42.126
ATOM     38  C   ???     1       4.930   4.525 -40.483
ATOM     39  H   ???     1       4.591   3.477 -40.408
ATOM     40  H   ???     1       5.774   4.662 -39.771
ATOM     41  O   ???     1       3.826   5.349 -40.081
ATOM     42  H   ???     1       4.200   6.184 -39.698
ATOM     43  H   ???     1       4.607   4.495 -42.654
ATOM     44  H   ???     1      13.632  11.344 -31.927
ATOM     45  C   ???     1      12.720  10.656 -31.972
ATOM     46  H   ???     1      12.875   9.950 -31.132
ATOM     47  H   ???     1      11.737  11.192 -31.789
ATOM     48  C   ???     1      12.894   9.821 -33.253
ATOM     49  O   ???     1      14.028   9.614 -33.692
```

```
ATOM     50  N   ???     1      11.797   9.223 -33.783
ATOM     51  H   ???     1      10.835   9.455 -33.493
ATOM     52  C   ???     1      11.955   8.139 -34.743
ATOM     53  H   ???     1      12.914   7.642 -34.537
ATOM     54  H   ???     1      11.124   7.433 -34.565
ATOM     55  C   ???     1      11.974   8.528 -36.230
ATOM     56  O   ???     1      12.987   8.407 -36.924
ATOM     57  N   ???     1      10.769   8.960 -36.723
ATOM     58  H   ???     1       9.931   8.817 -36.132
ATOM     59  C   ???     1      10.575   9.143 -38.148
ATOM     60  H   ???     1      11.571   9.264 -38.609
ATOM     61  H   ???     1      10.102   8.251 -38.598
ATOM     62  C   ???     1       9.680  10.299 -38.583
ATOM     63  O   ???     1       9.550  10.537 -39.790
ATOM     64  N   ???     1       9.050  11.004 -37.606
ATOM     65  H   ???     1       9.096  10.658 -36.639
ATOM     66  C   ???     1       8.099  12.061 -37.929
ATOM     67  H   ???     1       8.333  12.411 -38.945
ATOM     68  H   ???     1       7.043  11.659 -37.929
ATOM     69  C   ???     1       8.208  13.252 -36.959
ATOM     70  O   ???     1       8.477  14.391 -37.365
ATOM     71  N   ???     1       7.919  12.932 -35.677
ATOM     72  H   ???     1       7.751  11.946 -35.440
ATOM     73  C   ???     1       7.845  13.900 -34.573
ATOM     74  H   ???     1       6.992  14.579 -34.789
ATOM     75  C   ???     1       7.532  13.117 -33.289
ATOM     76  H   ???     1       8.316  12.350 -33.121
ATOM     77  H   ???     1       7.567  13.817 -32.432
ATOM     78  C   ???     1       6.156  12.424 -33.322
ATOM     79  H   ???     1       5.358  13.195 -33.292
ATOM     80  H   ???     1       6.019  11.855 -34.264
ATOM     81  C   ???     1       5.970  11.443 -32.164
ATOM     82  H   ???     1       6.652  10.577 -32.289
ATOM     83  H   ???     1       6.217  11.946 -31.207
ATOM     84  N   ???     1       4.575  10.993 -32.115
ATOM     85  H   ???     1       3.966  11.167 -32.929
ATOM     86  C   ???     1       3.996  10.357 -31.090
ATOM     87  N   ???     1       4.678  10.137 -29.955
ATOM     88  H   ???     1       5.628  10.505 -29.846
ATOM     89  H   ???     1       4.338   9.494 -29.233
ATOM     90  N   ???     1       2.713   9.940 -31.214
ATOM     91  H   ???     1       2.242  10.191 -32.094
ATOM     92  H   ???     1       2.106   9.946 -30.391
ATOM     93  H   ???     1       8.731  14.565 -34.480
ATOM     94  H   ???     1       8.405   7.460 -28.186
ATOM     95  C   ???     1       9.349   7.499 -28.749
ATOM     96  N   ???     1       9.550   6.767 -29.911
ATOM     97  C   ???     1      10.852   6.867 -30.198
ATOM     98  H   ???     1      11.354   6.417 -31.055
ATOM     99  N   ???     1      11.484   7.640 -29.281
ATOM    100  H   ???     1      12.492   7.877 -29.297
ATOM    101  C   ???     1      10.551   8.047 -28.348
ATOM    102  H   ???     1      10.823   8.714 -27.531
ATOM    103  O   ???     1      10.666   5.259 -25.638
ATOM    104  C   ???     1      10.949   4.627 -26.716
ATOM    105  O   ???     1      12.095   4.360 -27.156
ATOM    106  C   ???     1       9.735   4.236 -27.586
ATOM    107  H   ???     1       8.847   4.118 -26.940
ATOM    108  H   ???     1       9.543   5.120 -28.218
ATOM    109  C   ???     1       9.877   3.044 -28.535
ATOM    110  H   ???     1      10.864   3.075 -29.025
ATOM    111  H   ???     1       9.829   2.083 -27.985
ATOM    112  C   ???     1       8.806   3.075 -29.663
ATOM    113  O   ???     1       9.275   3.989 -30.687
ATOM    114  H   ???     1       9.879   3.365 -31.279
ATOM    115  C   ???     1       7.496   3.630 -29.096
ATOM    116  O   ???     1       7.133   4.779 -29.543
ATOM    117  O   ???     1       6.869   2.987 -28.223
ATOM    118  C   ???     1       8.555   1.715 -30.342
ATOM    119  H   ???     1       8.155   0.988 -29.614
ATOM    120  H   ???     1       7.788   1.875 -31.129
ATOM    121  C   ???     1       9.806   1.137 -31.034
ATOM    122  O   ???     1      10.551   2.014 -31.632
ATOM    123  O   ???     1       9.984  -0.094 -31.005
ATOM    124 FE   ???     1       5.889   6.434 -32.645
ATOM    125 MO   ???     1       8.037   5.712 -31.319
ATOM    126 FE   ???     1       7.788   5.100 -33.868
ATOM    127 FE   ???     1       8.010   7.723 -33.148
ATOM    128 FE   ???     1       4.833   7.262 -34.759
ATOM    129 FE   ???     1       6.803   6.120 -36.061
ATOM    130 FE   ???     1       5.342   7.875 -37.202
ATOM    131 FE   ???     1       6.972   8.557 -35.337
ATOM    132  C   ???     1       6.690   6.891 -34.315
ATOM    133  S   ???     1       7.671   7.694 -37.367
ATOM    134  S   ???     1       9.644   6.182 -33.013
ATOM    135  S   ???     1       4.614   5.864 -36.541
ATOM    136  S   ???     1       7.817   4.243 -35.998
ATOM    137  S   ???     1       8.554   9.649 -34.178
ATOM    138  S   ???     1       6.377   4.302 -32.321
ATOM    139  S   ???     1       4.810   9.364 -35.501
ATOM    140  S   ???     1       6.892   7.782 -31.186
ATOM    141  S   ???     1       3.717   6.762 -32.889
ATOM    142  N   ???     1       8.651   3.461 -33.715
ATOM    143  H   ???     1      10.346   2.470 -33.658
ATOM    144  N   ???     1       9.892   3.368 -33.883
ATOM    145  H   ???     1      10.495   4.214 -34.016
ATOM    146  O   ???     1       4.513   5.538 -29.470
ATOM    147  H   ???     1       5.467   5.266 -29.533
ATOM    148  H   ???     1       4.399   6.201 -30.189
ATOM    149  O   ???     1       1.081   8.392 -34.244
ATOM    150  H   ???     1       1.946   8.072 -33.886
ATOM    151  H   ???     1       0.567   8.684 -33.433
END
```

Fe6-HNNH2

```
REMARK Energies (QM/MM, QM+ptch) =  -17129.762036 -16290.139306
ATOM      1  H   ???     1       3.501   2.240 -29.686
ATOM      2  C   ???     1       3.912   2.095 -30.704
ATOM      3  H   ???     1       5.017   2.108 -30.632
ATOM      4  H   ???     1       3.597   1.114 -31.094
ATOM      5  N   ???     1       3.492   3.151 -31.615
ATOM      6  H   ???     1       3.966   4.059 -31.499
ATOM      7  C   ???     1       2.859   2.936 -32.781
ATOM      8  N   ???     1       2.000   1.892 -32.911
ATOM      9  H   ???     1       1.823   1.283 -32.110
ATOM     10  H   ???     1       1.798   1.506 -33.844
ATOM     11  N   ???     1       3.076   3.756 -33.812
ATOM     12  H   ???     1       3.640   4.622 -33.669
ATOM     13  H   ???     1       2.421   3.787 -34.598
ATOM     14  H   ???     1       7.275   1.561 -41.620
ATOM     15  C   ???     1       7.581   2.027 -40.686
ATOM     16  N   ???     1       8.439   3.110 -40.699
ATOM     17  C   ???     1       8.512   3.508 -39.434
ATOM     18  H   ???     1       9.080   4.350 -39.039
ATOM     19  N   ???     1       7.746   2.727 -38.624
ATOM     20  H   ???     1       7.592   2.931 -37.620
ATOM     21  C   ???     1       7.127   1.781 -39.406
ATOM     22  H   ???     1       6.431   1.052 -38.991
ATOM     23  H   ???     1       2.977   9.557 -40.117
ATOM     24  C   ???     1       3.101   8.705 -39.421
ATOM     25  H   ???     1       2.511   7.838 -39.764
ATOM     26  H   ???     1       2.784   9.023 -38.414
ATOM     27  S   ???     1       4.916   8.306 -39.415
ATOM     28  H   ???     1       6.637   8.643 -42.923
ATOM     29  C   ???     1       7.435   7.842 -42.957
ATOM     30  H   ???     1       8.006   8.022 -43.881
ATOM     31  H   ???     1       8.092   7.980 -42.081
ATOM     32  C   ???     1       6.837   6.419 -43.008
ATOM     33  O   ???     1       7.261   5.638 -43.870
ATOM     34  N   ???     1       5.923   6.124 -42.063
ATOM     35  H   ???     1       5.589   6.846 -41.408
ATOM     36  C   ???     1       5.390   4.763 -41.927
ATOM     37  H   ???     1       6.246   4.094 -42.129
ATOM     38  C   ???     1       4.924   4.520 -40.488
ATOM     39  H   ???     1       4.591   3.469 -40.417
ATOM     40  H   ???     1       5.762   4.661 -39.772
ATOM     41  O   ???     1       3.813   5.336 -40.093
ATOM     42  H   ???     1       4.180   6.175 -39.716
ATOM     43  H   ???     1       4.607   4.494 -42.656
ATOM     44  H   ???     1      13.632  11.342 -31.927
ATOM     45  C   ???     1      12.719  10.655 -31.970
ATOM     46  H   ???     1      12.873   9.949 -31.129
ATOM     47  H   ???     1      11.736  11.192 -31.788
ATOM     48  C   ???     1      12.891   9.820 -33.252
ATOM     49  O   ???     1      14.022   9.625 -33.702
ATOM     50  N   ???     1      11.796   9.212 -33.780
ATOM     51  H   ???     1      10.835   9.414 -33.467
ATOM     52  C   ???     1      11.970   8.131 -34.742
ATOM     53  H   ???     1      12.933   7.644 -34.534
ATOM     54  H   ???     1      11.151   7.411 -34.569
ATOM     55  C   ???     1      11.988   8.528 -36.226
ATOM     56  O   ???     1      12.995   8.405 -36.925
ATOM     57  N   ???     1      10.783   8.973 -36.714
ATOM     58  H   ???     1       9.946   8.824 -36.126
ATOM     59  C   ???     1      10.594   9.155 -38.140
ATOM     60  H   ???     1      11.590   9.305 -38.594
ATOM     61  H   ???     1      10.155   8.253 -38.603
ATOM     62  C   ???     1       9.672  10.289 -38.577
ATOM     63  O   ???     1       9.519  10.505 -39.785
ATOM     64  N   ???     1       9.054  11.007 -37.603
ATOM     65  H   ???     1       9.128  10.688 -36.629
ATOM     66  C   ???     1       8.100  12.062 -37.928
ATOM     67  H   ???     1       8.333  12.411 -38.944
ATOM     68  H   ???     1       7.043  11.659 -37.929
ATOM     69  C   ???     1       8.207  13.252 -36.958
ATOM     70  O   ???     1       8.476  14.390 -37.363
ATOM     71  N   ???     1       7.918  12.931 -35.676
ATOM     72  H   ???     1       7.746  11.945 -35.440
ATOM     73  C   ???     1       7.844  13.899 -34.572
ATOM     74  H   ???     1       6.991  14.578 -34.787
ATOM     75  C   ???     1       7.531  13.118 -33.287
ATOM     76  H   ???     1       8.319  12.357 -33.112
ATOM     77  H   ???     1       7.558  13.822 -32.433
ATOM     78  C   ???     1       6.160  12.415 -33.322
ATOM     79  H   ???     1       5.357  13.183 -33.323
ATOM     80  H   ???     1       6.040  11.819 -34.248
ATOM     81  C   ???     1       5.962  11.467 -32.138
ATOM     82  H   ???     1       6.654  10.605 -32.224
ATOM     83  H   ???     1       6.185  12.001 -31.192
ATOM     84  N   ???     1       4.572  10.999 -32.103
ATOM     85  H   ???     1       3.962  11.184 -32.913
ATOM     86  C   ???     1       3.990  10.365 -31.078
ATOM     87  N   ???     1       4.668  10.157 -29.939
ATOM     88  H   ???     1       5.617  10.525 -29.828
ATOM     89  H   ???     1       4.328   9.514 -29.216
ATOM     90  N   ???     1       2.710   9.939 -31.207
ATOM     91  H   ???     1       2.242  10.189 -32.089
ATOM     92  H   ???     1       2.100   9.949 -30.387
ATOM     93  H   ???     1       8.731  14.564 -34.480
ATOM     94  H   ???     1       8.402   7.472 -28.174
ATOM     95  C   ???     1       9.349   7.530 -28.717
ATOM     96  N   ???     1       9.553   6.787 -29.864
ATOM     97  C   ???     1      10.861   6.855 -30.131
ATOM     98  H   ???     1      11.373   6.366 -30.960
ATOM     99  N   ???     1      11.491   7.632 -29.217
ATOM    100  H   ???     1      12.501   7.858 -29.230
ATOM    101  C   ???     1      10.552   8.066 -28.303
ATOM    102  H   ???     1      10.820   8.743 -27.492
ATOM    103  O   ???     1      10.649   5.254 -25.648
ATOM    104  C   ???     1      10.943   4.629 -26.731
```

```
ATOM    105  O   ???     1      12.096   4.386 -27.164
ATOM    106  C   ???     1       9.734   4.220 -27.602
ATOM    107  H   ???     1       8.872   4.017 -26.942
ATOM    108  H   ???     1       9.478   5.131 -28.171
ATOM    109  C   ???     1       9.906   3.098 -28.634
ATOM    110  H   ???     1      10.884   3.196 -29.134
ATOM    111  H   ???     1       9.890   2.101 -28.147
ATOM    112  C   ???     1       8.827   3.170 -29.762
ATOM    113  O   ???     1       9.233   4.133 -30.714
ATOM    114  H   ???     1       9.387   3.678 -32.330
ATOM    115  C   ???     1       7.499   3.641 -29.121
ATOM    116  O   ???     1       7.081   4.794 -29.541
ATOM    117  O   ???     1       6.894   2.983 -28.246
ATOM    118  C   ???     1       8.557   1.789 -30.427
ATOM    119  H   ???     1       8.064   1.129 -29.690
ATOM    120  H   ???     1       7.828   1.970 -31.246
ATOM    121  C   ???     1       9.770   1.045 -31.037
ATOM    122  O   ???     1      10.513   1.725 -31.831
ATOM    123  O   ???     1       9.910  -0.172 -30.782
ATOM    124 FE   ???     1       7.927   7.753 -33.079
ATOM    125 MO   ???     1       8.041   5.728 -31.251
ATOM    126 FE   ???     1       7.005   8.481 -35.317
ATOM    127 FE   ???     1       5.828   6.417 -32.614
ATOM    128 FE   ???     1       8.008   5.129 -33.861
ATOM    129 FE   ???     1       6.843   6.016 -36.043
ATOM    130 FE   ???     1       5.506   7.801 -37.288
ATOM    131 FE   ???     1       4.883   7.142 -34.820
ATOM    132  C   ???     1       6.755   6.819 -34.277
ATOM    133  S   ???     1       7.727   7.684 -37.352
ATOM    134  S   ???     1       9.653   6.370 -32.894
ATOM    135  S   ???     1       4.732   5.729 -36.623
ATOM    136  S   ???     1       7.969   4.182 -35.927
ATOM    137  S   ???     1       8.512   9.633 -34.179
ATOM    138  S   ???     1       6.481   4.277 -32.373
ATOM    139  S   ???     1       4.928   9.288 -35.600
ATOM    140  S   ???     1       6.773   7.759 -31.131
ATOM    141  S   ???     1       3.678   6.751 -32.962
ATOM    142  N   ???     1       9.036   3.692 -33.340
ATOM    143  H   ???     1       9.772   1.875 -33.241
ATOM    144  N   ???     1       9.223   2.539 -33.865
ATOM    145  H   ???     1       8.900   2.449 -34.845
ATOM    146  O   ???     1       4.494   5.524 -29.473
ATOM    147  H   ???     1       5.453   5.245 -29.496
ATOM    148  H   ???     1       4.446   6.280 -30.099
ATOM    149  O   ???     1       1.065   8.393 -34.246
ATOM    150  H   ???     1       1.916   8.050 -33.878
ATOM    151  H   ???     1       0.547   8.690 -33.440
END

Fe3/7Fe4/5
REMARK Energies (QM/MM, QM+ptch) =  -17129.751918 -16290.127014
ATOM      1  H   ???     1       3.501   2.238 -29.687
ATOM      2  C   ???     1       3.913   2.084 -30.706
ATOM      3  H   ???     1       5.006   2.241 -30.656
ATOM      4  H   ???     1       3.719   1.046 -31.024
ATOM      5  N   ???     1       3.363   2.998 -31.696
ATOM      6  H   ???     1       3.840   3.909 -31.813
ATOM      7  C   ???     1       2.668   2.579 -32.771
ATOM      8  N   ???     1       1.853   1.500 -32.697
ATOM      9  H   ???     1       1.706   1.011 -31.810
ATOM     10  H   ???     1       1.629   1.009 -33.571
ATOM     11  N   ???     1       2.795   3.222 -33.928
ATOM     12  H   ???     1       3.281   4.166 -33.903
ATOM     13  H   ???     1       2.096   3.087 -34.665
ATOM     14  H   ???     1       7.277   1.566 -41.610
ATOM     15  C   ???     1       7.583   2.040 -40.669
ATOM     16  N   ???     1       8.437   3.127 -40.697
ATOM     17  C   ???     1       8.514   3.543 -39.439
ATOM     18  H   ???     1       9.084   4.391 -39.061
ATOM     19  N   ???     1       7.754   2.775 -38.612
ATOM     20  H   ???     1       7.621   2.979 -37.604
ATOM     21  C   ???     1       7.136   1.812 -39.380
ATOM     22  H   ???     1       6.447   1.086 -38.951
ATOM     23  H   ???     1       2.984   9.554 -40.118
ATOM     24  C   ???     1       3.119   8.698 -39.423
ATOM     25  H   ???     1       2.477   7.858 -39.738
ATOM     26  H   ???     1       2.865   9.023 -38.400
ATOM     27  S   ???     1       4.912   8.190 -39.469
ATOM     28  H   ???     1       6.638   8.643 -42.924
ATOM     29  C   ???     1       7.437   7.843 -42.960
ATOM     30  H   ???     1       8.009   8.029 -43.882
ATOM     31  H   ???     1       8.093   7.981 -42.082
ATOM     32  C   ???     1       6.842   6.419 -43.017
ATOM     33  O   ???     1       7.270   5.643 -43.883
ATOM     34  N   ???     1       5.928   6.121 -42.075
ATOM     35  H   ???     1       5.590   6.842 -41.418
ATOM     36  C   ???     1       5.398   4.759 -41.938
ATOM     37  H   ???     1       6.255   4.091 -42.143
ATOM     38  C   ???     1       4.926   4.513 -40.502
ATOM     39  H   ???     1       4.628   3.453 -40.424
ATOM     40  H   ???     1       5.751   4.691 -39.777
ATOM     41  O   ???     1       3.784   5.293 -40.131
ATOM     42  H   ???     1       4.123   6.161 -39.790
ATOM     43  H   ???     1       4.612   4.493 -42.662
ATOM     44  H   ???     1      13.631  11.346 -31.927
ATOM     45  C   ???     1      12.722  10.655 -31.975
ATOM     46  H   ???     1      12.877   9.949 -31.135
ATOM     47  H   ???     1      11.738  11.191 -31.790
ATOM     48  C   ???     1      12.921   9.824 -33.259
ATOM     49  O   ???     1      14.067   9.636 -33.675
ATOM     50  N   ???     1      11.845   9.215 -33.814
ATOM     51  H   ???     1      10.870   9.418 -33.540
ATOM     52  C   ???     1      12.037   8.138 -34.779
ATOM     53  H   ???     1      13.007   7.669 -34.567
ATOM     54  H   ???     1      11.226   7.408 -34.603
```

```
ATOM     55  C   ???     1      12.048   8.515 -36.274
ATOM     56  O   ???     1      13.046   8.345 -36.981
ATOM     57  N   ???     1      10.851   8.975 -36.749
ATOM     58  H   ???     1      10.039   8.917 -36.098
ATOM     59  C   ???     1      10.615   9.147 -38.167
ATOM     60  H   ???     1      11.593   9.268 -38.666
ATOM     61  H   ???     1      10.130   8.251 -38.598
ATOM     62  C   ???     1       9.703  10.299 -38.587
ATOM     63  O   ???     1       9.582  10.563 -39.791
ATOM     64  N   ???     1       9.050  10.975 -37.603
ATOM     65  H   ???     1       9.046  10.581 -36.649
ATOM     66  C   ???     1       8.106  12.041 -37.923
ATOM     67  H   ???     1       8.346  12.394 -38.936
ATOM     68  H   ???     1       7.046  11.652 -37.927
ATOM     69  C   ???     1       8.206  13.228 -36.950
ATOM     70  O   ???     1       8.439  14.374 -37.359
ATOM     71  N   ???     1       7.927  12.912 -35.663
ATOM     72  H   ???     1       7.868  11.919 -35.391
ATOM     73  C   ???     1       7.854  13.912 -34.591
ATOM     74  H   ???     1       7.010  14.592 -34.834
ATOM     75  C   ???     1       7.538  13.196 -33.275
ATOM     76  H   ???     1       8.301  12.415 -33.078
ATOM     77  H   ???     1       7.595  13.934 -32.452
ATOM     78  C   ???     1       6.151  12.538 -33.268
ATOM     79  H   ???     1       5.373  13.308 -33.447
ATOM     80  H   ???     1       6.067  11.801 -34.093
ATOM     81  C   ???     1       5.866  11.835 -31.942
ATOM     82  H   ???     1       6.629  11.054 -31.746
ATOM     83  H   ???     1       5.935  12.557 -31.103
ATOM     84  N   ???     1       4.533  11.221 -31.988
ATOM     85  H   ???     1       3.896  11.462 -32.765
ATOM     86  C   ???     1       4.009  10.423 -31.040
ATOM     87  N   ???     1       4.704  10.149 -29.932
ATOM     88  H   ???     1       5.606  10.606 -29.761
ATOM     89  H   ???     1       4.415   9.420 -29.268
ATOM     90  N   ???     1       2.792   9.876 -31.266
ATOM     91  H   ???     1       2.280  10.194 -32.103
ATOM     92  H   ???     1       2.213   9.589 -30.476
ATOM     93  H   ???     1       8.736  14.572 -34.492
ATOM     94  H   ???     1       8.405   7.460 -28.187
ATOM     95  C   ???     1       9.347   7.498 -28.754
ATOM     96  N   ???     1       9.531   6.765 -29.918
ATOM     97  C   ???     1      10.829   6.856 -30.218
ATOM     98  H   ???     1      11.317   6.401 -31.081
ATOM     99  N   ???     1      11.478   7.623 -29.306
ATOM    100  H   ???     1      12.487   7.855 -29.331
ATOM    101  C   ???     1      10.557   8.036 -28.364
ATOM    102  H   ???     1      10.843   8.701 -27.549
ATOM    103  O   ???     1      10.658   5.252 -25.646
ATOM    104  C   ???     1      10.938   4.622 -26.727
ATOM    105  O   ???     1      12.084   4.349 -27.163
ATOM    106  C   ???     1       9.721   4.245 -27.601
ATOM    107  H   ???     1       8.837   4.112 -26.952
ATOM    108  H   ???     1       9.525   5.140 -28.216
ATOM    109  C   ???     1       9.855   3.074 -28.578
ATOM    110  H   ???     1      10.842   3.119 -29.069
ATOM    111  H   ???     1       9.808   2.102 -28.045
ATOM    112  C   ???     1       8.775   3.140 -29.703
ATOM    113  O   ???     1       9.218   4.034 -30.715
ATOM    114  H   ???     1       9.962   3.095 -31.499
ATOM    115  C   ???     1       7.458   3.639 -29.093
ATOM    116  O   ???     1       7.057   4.771 -29.544
ATOM    117  O   ???     1       6.858   2.978 -28.212
ATOM    118  C   ???     1       8.487   1.771 -30.376
ATOM    119  H   ???     1       8.114   1.029 -29.650
ATOM    120  H   ???     1       7.697   1.948 -31.136
ATOM    121  C   ???     1       9.706   1.215 -31.095
ATOM    122  O   ???     1      10.405   2.135 -31.760
ATOM    123  O   ???     1      10.000   0.021 -31.072
ATOM    124 FE   ???     1       6.873   8.455 -35.278
ATOM    125 MO   ???     1       8.004   5.668 -31.307
ATOM    126 FE   ???     1       4.835   7.079 -34.805
ATOM    127 FE   ???     1       5.741   6.445 -32.630
ATOM    128 FE   ???     1       7.772   7.800 -33.086
ATOM    129 FE   ???     1       5.425   7.634 -37.351
ATOM    130 FE   ???     1       6.858   5.978 -36.086
ATOM    131 FE   ???     1       7.751   5.252 -33.849
ATOM    132  C   ???     1       6.691   6.748 -34.351
ATOM    133  S   ???     1       7.685   7.715 -37.286
ATOM    134  S   ???     1       9.530   6.313 -33.029
ATOM    135  S   ???     1       4.715   5.631 -36.581
ATOM    136  S   ???     1       8.010   4.138 -35.694
ATOM    137  S   ???     1       8.701   9.512 -34.394
ATOM    138  S   ???     1       6.397   4.256 -32.386
ATOM    139  S   ???     1       4.665   9.088 -35.905
ATOM    140  S   ???     1       6.786   7.623 -31.082
ATOM    141  S   ???     1       3.570   6.055 -33.150
ATOM    142  N   ???     1       5.110   8.178 -33.215
ATOM    143  H   ???     1       4.319   8.673 -32.785
ATOM    144  N   ???     1       6.248   8.946 -33.472
ATOM    145  H   ???     1       6.166   9.928 -33.198
ATOM    146  O   ???     1       4.471   5.580 -29.439
ATOM    147  H   ???     1       5.411   5.268 -29.521
ATOM    148  H   ???     1       4.280   5.996 -30.311
ATOM    149  O   ???     1       1.133   8.365 -34.201
ATOM    150  H   ???     1       1.908   7.846 -33.863
ATOM    151  H   ???     1       0.611   8.636 -33.391
END

Fe2(trans)
REMARK Energies (QM/MM, QM+ptch) =  -17129.766955 -16290.137760
ATOM      1  H   ???     1       3.501   2.241 -29.687
ATOM      2  C   ???     1       3.909   2.098 -30.708
ATOM      3  H   ???     1       5.013   2.109 -30.637
ATOM      4  H   ???     1       3.592   1.118 -31.098
```

ATOM	5	N	???	1	3.493	3.154	-31.620
ATOM	6	H	???	1	3.981	4.057	-31.516
ATOM	7	C	???	1	2.859	2.939	-32.785
ATOM	8	N	???	1	1.997	1.896	-32.913
ATOM	9	H	???	1	1.821	1.286	-32.112
ATOM	10	H	???	1	1.794	1.510	-33.846
ATOM	11	N	???	1	3.077	3.759	-33.815
ATOM	12	H	???	1	3.633	4.630	-33.661
ATOM	13	H	???	1	2.419	3.793	-34.600
ATOM	14	H	???	1	7.285	1.552	-41.606
ATOM	15	C	???	1	7.615	1.993	-40.657
ATOM	16	N	???	1	8.576	2.984	-40.667
ATOM	17	C	???	1	8.816	3.256	-39.393
ATOM	18	H	???	1	9.514	3.996	-39.003
ATOM	19	N	???	1	8.045	2.494	-38.564
ATOM	20	H	???	1	7.996	2.635	-37.537
ATOM	21	C	???	1	7.254	1.685	-39.358
ATOM	22	H	???	1	6.524	0.996	-38.934
ATOM	23	H	???	1	2.986	9.555	-40.114
ATOM	24	C	???	1	3.127	8.704	-39.412
ATOM	25	H	???	1	2.585	7.812	-39.772
ATOM	26	H	???	1	2.759	9.010	-38.419
ATOM	27	S	???	1	4.964	8.376	-39.335
ATOM	28	H	???	1	6.631	8.637	-42.917
ATOM	29	C	???	1	7.422	7.828	-42.944
ATOM	30	H	???	1	7.988	7.991	-43.874
ATOM	31	H	???	1	8.088	7.976	-42.076
ATOM	32	C	???	1	6.816	6.407	-42.980
ATOM	33	O	???	1	7.231	5.626	-43.847
ATOM	34	N	???	1	5.901	6.112	-42.035
ATOM	35	H	???	1	5.611	6.816	-41.342
ATOM	36	C	???	1	5.368	4.750	-41.923
ATOM	37	H	???	1	6.224	4.078	-42.113
ATOM	38	C	???	1	4.828	4.450	-40.520
ATOM	39	H	???	1	4.349	3.455	-40.561
ATOM	40	H	???	1	5.647	4.398	-39.784
ATOM	41	O	???	1	3.819	5.366	-40.069
ATOM	42	H	???	1	4.267	6.157	-39.671
ATOM	43	H	???	1	4.600	4.490	-42.659
ATOM	44	H	???	1	13.633	11.343	-31.926
ATOM	45	C	???	1	12.721	10.656	-31.968
ATOM	46	H	???	1	12.875	9.952	-31.126
ATOM	47	H	???	1	11.737	11.192	-31.787
ATOM	48	C	???	1	12.895	9.816	-33.248
ATOM	49	O	???	1	14.026	9.621	-33.697
ATOM	50	N	???	1	11.798	9.207	-33.762
ATOM	51	H	???	1	10.837	9.426	-33.455
ATOM	52	C	???	1	11.952	8.124	-34.724
ATOM	53	H	???	1	12.903	7.615	-34.518
ATOM	54	H	???	1	11.115	7.424	-34.553
ATOM	55	C	???	1	11.980	8.530	-36.206
ATOM	56	O	???	1	12.991	8.409	-36.902
ATOM	57	N	???	1	10.780	8.988	-36.696
ATOM	58	H	???	1	9.936	8.822	-36.122
ATOM	59	C	???	1	10.593	9.161	-38.125
ATOM	60	H	???	1	11.590	9.301	-38.578
ATOM	61	H	???	1	10.148	8.258	-38.580
ATOM	62	C	???	1	9.677	10.296	-38.573
ATOM	63	O	???	1	9.517	10.495	-39.783
ATOM	64	N	???	1	9.073	11.034	-37.604
ATOM	65	H	???	1	9.139	10.708	-36.634
ATOM	66	C	???	1	8.103	12.072	-37.930
ATOM	67	H	???	1	8.330	12.426	-38.946
ATOM	68	H	???	1	7.045	11.663	-37.930
ATOM	69	C	???	1	8.205	13.260	-36.958
ATOM	70	O	???	1	8.478	14.400	-37.359
ATOM	71	N	???	1	7.915	12.937	-35.677
ATOM	72	H	???	1	7.727	11.953	-35.447
ATOM	73	C	???	1	7.844	13.903	-34.571
ATOM	74	H	???	1	6.992	14.584	-34.786
ATOM	75	C	???	1	7.533	13.125	-33.285
ATOM	76	H	???	1	8.316	12.358	-33.112
ATOM	77	H	???	1	7.570	13.831	-32.432
ATOM	78	C	???	1	6.158	12.434	-33.312
ATOM	79	H	???	1	5.359	13.205	-33.308
ATOM	80	H	???	1	6.027	11.840	-34.239
ATOM	81	C	???	1	5.960	11.482	-32.131
ATOM	82	H	???	1	6.650	10.619	-32.224
ATOM	83	H	???	1	6.188	12.011	-31.183
ATOM	84	N	???	1	4.568	11.023	-32.096
ATOM	85	H	???	1	3.964	11.194	-32.914
ATOM	86	C	???	1	3.995	10.360	-31.085
ATOM	87	N	???	1	4.680	10.130	-29.954
ATOM	88	H	???	1	5.623	10.512	-29.837
ATOM	89	H	???	1	4.343	9.480	-29.237
ATOM	90	N	???	1	2.718	9.929	-31.221
ATOM	91	H	???	1	2.247	10.194	-32.097
ATOM	92	H	???	1	2.110	9.911	-30.399
ATOM	93	H	???	1	8.731	14.566	-34.480
ATOM	94	H	???	1	8.407	7.459	-28.187
ATOM	95	C	???	1	9.352	7.497	-28.749
ATOM	96	N	???	1	9.563	6.751	-29.900
ATOM	97	C	???	1	10.864	6.856	-30.184
ATOM	98	H	???	1	11.370	6.393	-31.034
ATOM	99	N	???	1	11.489	7.644	-29.276
ATOM	100	H	???	1	12.496	7.886	-29.293
ATOM	101	C	???	1	10.551	8.056	-28.350
ATOM	102	H	???	1	10.816	8.735	-27.541
ATOM	103	O	???	1	10.667	5.255	-25.629
ATOM	104	C	???	1	10.946	4.622	-26.709
ATOM	105	O	???	1	12.091	4.350	-27.148
ATOM	106	C	???	1	9.730	4.240	-27.578
ATOM	107	H	???	1	8.839	4.135	-26.933
ATOM	108	H	???	1	9.550	5.120	-28.219
ATOM	109	C	???	1	9.858	3.043	-28.521

```
ATOM    110  H   ???     1      10.850   3.065 -29.003
ATOM    111  H   ???     1       9.794   2.084 -27.967
ATOM    112  C   ???     1       8.797   3.110 -29.661
ATOM    113  O   ???     1       9.282   3.993 -30.662
ATOM    114  H   ???     1       9.994   3.075 -31.428
ATOM    115  C   ???     1       7.474   3.631 -29.083
ATOM    116  O   ???     1       7.090   4.765 -29.550
ATOM    117  O   ???     1       6.851   2.980 -28.212
ATOM    118  C   ???     1       8.508   1.742 -30.334
ATOM    119  H   ???     1       8.133   0.999 -29.610
ATOM    120  H   ???     1       7.718   1.918 -31.094
ATOM    121  C   ???     1       9.723   1.183 -31.060
ATOM    122  O   ???     1      10.430   2.097 -31.715
ATOM    123  O   ???     1       9.996  -0.018 -31.050
ATOM    124 FE   ???     1       5.903   6.325 -32.619
ATOM    125 MO   ???     1       8.089   5.629 -31.308
ATOM    126 FE   ???     1       7.776   5.238 -33.917
ATOM    127 FE   ???     1       8.063   7.635 -33.073
ATOM    128 FE   ???     1       4.840   7.240 -34.680
ATOM    129 FE   ???     1       6.961   6.111 -36.197
ATOM    130 FE   ???     1       5.342   7.983 -37.095
ATOM    131 FE   ???     1       6.908   8.644 -35.101
ATOM    132  C   ???     1       6.735   6.872 -34.305
ATOM    133  S   ???     1       7.691   8.082 -37.135
ATOM    134  S   ???     1       9.691   6.035 -33.009
ATOM    135  S   ???     1       4.643   5.933 -36.503
ATOM    136  S   ???     1       7.985   4.117 -35.753
ATOM    137  S   ???     1       8.565   9.626 -33.964
ATOM    138  S   ???     1       6.531   4.164 -32.410
ATOM    139  S   ???     1       4.736   9.376 -35.350
ATOM    140  S   ???     1       6.880   7.674 -31.154
ATOM    141  S   ???     1       3.726   6.688 -32.828
ATOM    142  N   ???     1       7.052   5.449 -38.020
ATOM    143  H   ???     1       6.788   4.449 -38.087
ATOM    144  N   ???     1       7.303   5.918 -39.158
ATOM    145  H   ???     1       7.537   6.931 -39.000
ATOM    146  O   ???     1       4.494   5.525 -29.479
ATOM    147  H   ???     1       5.452   5.259 -29.529
ATOM    148  H   ???     1       4.390   6.209 -30.180
ATOM    149  O   ???     1       1.094   8.389 -34.237
ATOM    150  H   ???     1       1.964   8.083 -33.880
ATOM    151  H   ???     1       0.579   8.681 -33.428
END

FE6(Trans)
REMARK Energies (QM/MM, QM+ptch) =  -17129.761127 -16290.136624
ATOM      1  H   ???     1       3.499   2.241 -29.688
ATOM      2  C   ???     1       3.903   2.099 -30.709
ATOM      3  H   ???     1       5.008   2.105 -30.645
ATOM      4  H   ???     1       3.578   1.122 -31.102
ATOM      5  N   ???     1       3.485   3.163 -31.611
ATOM      6  H   ???     1       3.977   4.063 -31.495
ATOM      7  C   ???     1       2.865   2.960 -32.787
ATOM      8  N   ???     1       2.006   1.917 -32.933
ATOM      9  H   ???     1       1.823   1.301 -32.140
ATOM     10  H   ???     1       1.806   1.541 -33.871
ATOM     11  N   ???     1       3.092   3.792 -33.805
ATOM     12  H   ???     1       3.650   4.660 -33.644
ATOM     13  H   ???     1       2.447   3.830 -34.600
ATOM     14  H   ???     1       7.277   1.561 -41.617
ATOM     15  C   ???     1       7.587   2.025 -40.682
ATOM     16  N   ???     1       8.442   3.111 -40.695
ATOM     17  C   ???     1       8.530   3.498 -39.428
ATOM     18  H   ???     1       9.095   4.344 -39.036
ATOM     19  N   ???     1       7.780   2.707 -38.614
ATOM     20  H   ???     1       7.625   2.910 -37.609
ATOM     21  C   ???     1       7.151   1.766 -39.396
ATOM     22  H   ???     1       6.462   1.033 -38.979
ATOM     23  H   ???     1       2.981   9.557 -40.119
ATOM     24  C   ???     1       3.113   8.706 -39.423
ATOM     25  H   ???     1       2.536   7.832 -39.770
ATOM     26  H   ???     1       2.781   9.021 -38.420
ATOM     27  S   ???     1       4.940   8.334 -39.404
ATOM     28  H   ???     1       6.637   8.643 -42.923
ATOM     29  C   ???     1       7.435   7.841 -42.957
ATOM     30  H   ???     1       8.006   8.021 -43.881
ATOM     31  H   ???     1       8.092   7.980 -42.081
ATOM     32  C   ???     1       6.836   6.419 -43.008
ATOM     33  O   ???     1       7.257   5.639 -43.871
ATOM     34  N   ???     1       5.922   6.126 -42.062
ATOM     35  H   ???     1       5.600   6.846 -41.399
ATOM     36  C   ???     1       5.389   4.765 -41.923
ATOM     37  H   ???     1       6.245   4.095 -42.125
ATOM     38  C   ???     1       4.928   4.527 -40.482
ATOM     39  H   ???     1       4.589   3.478 -40.408
ATOM     40  H   ???     1       5.771   4.664 -39.771
ATOM     41  O   ???     1       3.823   5.350 -40.081
ATOM     42  H   ???     1       4.198   6.188 -39.704
ATOM     43  H   ???     1       4.606   4.495 -42.653
ATOM     44  H   ???     1      13.632  11.344 -31.927
ATOM     45  C   ???     1      12.720  10.656 -31.971
ATOM     46  H   ???     1      12.875   9.950 -31.130
ATOM     47  H   ???     1      11.737  11.192 -31.788
ATOM     48  C   ???     1      12.897   9.820 -33.253
ATOM     49  O   ???     1      14.033   9.620 -33.691
ATOM     50  N   ???     1      11.804   9.216 -33.782
ATOM     51  H   ???     1      10.839   9.444 -33.495
ATOM     52  C   ???     1      11.969   8.134 -34.743
ATOM     53  H   ???     1      12.930   7.644 -34.540
ATOM     54  H   ???     1      11.146   7.419 -34.565
ATOM     55  C   ???     1      11.982   8.527 -36.230
ATOM     56  O   ???     1      12.991   8.405 -36.929
ATOM     57  N   ???     1      10.775   8.964 -36.716
ATOM     58  H   ???     1       9.940   8.823 -36.121
ATOM     59  C   ???     1      10.577   9.143 -38.142
```

```
ATOM     60  H   ???     1      11.571   9.266 -38.606
ATOM     61  H   ???     1      10.106   8.249 -38.589
ATOM     62  C   ???     1       9.677  10.294 -38.578
ATOM     63  O   ???     1       9.539  10.524 -39.786
ATOM     64  N   ???     1       9.052  11.005 -37.602
ATOM     65  H   ???     1       9.103  10.664 -36.634
ATOM     66  C   ???     1       8.100  12.061 -37.927
ATOM     67  H   ???     1       8.335  12.411 -38.943
ATOM     68  H   ???     1       7.043  11.659 -37.929
ATOM     69  C   ???     1       8.206  13.251 -36.956
ATOM     70  O   ???     1       8.472  14.391 -37.363
ATOM     71  N   ???     1       7.918  12.931 -35.675
ATOM     72  H   ???     1       7.757  11.943 -35.437
ATOM     73  C   ???     1       7.845  13.897 -34.570
ATOM     74  H   ???     1       6.991  14.577 -34.785
ATOM     75  C   ???     1       7.534  13.113 -33.287
ATOM     76  H   ???     1       8.317  12.344 -33.123
ATOM     77  H   ???     1       7.572  13.810 -32.428
ATOM     78  C   ???     1       6.156  12.422 -33.319
ATOM     79  H   ???     1       5.359  13.194 -33.274
ATOM     80  H   ???     1       6.013  11.865 -34.267
ATOM     81  C   ???     1       5.974  11.427 -32.172
ATOM     82  H   ???     1       6.647  10.558 -32.316
ATOM     83  H   ???     1       6.234  11.915 -31.211
ATOM     84  N   ???     1       4.575  10.988 -32.115
ATOM     85  H   ???     1       3.967  11.161 -32.929
ATOM     86  C   ???     1       3.994  10.359 -31.087
ATOM     87  N   ???     1       4.675  10.142 -29.951
ATOM     88  H   ???     1       5.628  10.500 -29.846
ATOM     89  H   ???     1       4.333   9.500 -29.229
ATOM     90  N   ???     1       2.708   9.946 -31.209
ATOM     91  H   ???     1       2.239  10.200 -32.089
ATOM     92  H   ???     1       2.103   9.961 -30.385
ATOM     93  H   ???     1       8.732  14.564 -34.479
ATOM     94  H   ???     1       8.405   7.467 -28.180
ATOM     95  C   ???     1       9.351   7.518 -28.733
ATOM     96  N   ???     1       9.554   6.785 -29.891
ATOM     97  C   ???     1      10.856   6.881 -30.175
ATOM     98  H   ???     1      11.361   6.422 -31.027
ATOM     99  N   ???     1      11.487   7.656 -29.260
ATOM    100  H   ???     1      12.495   7.893 -29.277
ATOM    101  C   ???     1      10.552   8.067 -28.330
ATOM    102  H   ???     1      10.821   8.741 -27.517
ATOM    103  O   ???     1      10.670   5.260 -25.619
ATOM    104  C   ???     1      10.935   4.638 -26.708
ATOM    105  O   ???     1      12.074   4.358 -27.158
ATOM    106  C   ???     1       9.709   4.278 -27.573
ATOM    107  H   ???     1       8.816   4.192 -26.929
ATOM    108  H   ???     1       9.548   5.157 -28.221
ATOM    109  C   ???     1       9.826   3.078 -28.510
ATOM    110  H   ???     1      10.826   3.088 -28.974
ATOM    111  H   ???     1       9.747   2.122 -27.952
ATOM    112  C   ???     1       8.774   3.144 -29.660
ATOM    113  O   ???     1       9.264   4.034 -30.657
ATOM    114  H   ???     1      10.235   3.019 -31.189
ATOM    115  C   ???     1       7.448   3.653 -29.082
ATOM    116  O   ???     1       7.064   4.794 -29.525
ATOM    117  O   ???     1       6.831   2.984 -28.218
ATOM    118  C   ???     1       8.513   1.768 -30.329
ATOM    119  H   ???     1       8.061   1.047 -29.626
ATOM    120  H   ???     1       7.789   1.936 -31.155
ATOM    121  C   ???     1       9.774   1.164 -30.927
ATOM    122  O   ???     1      10.640   2.062 -31.407
ATOM    123  O   ???     1       9.976  -0.045 -30.974
ATOM    124 FE   ???     1       8.005   7.716 -33.087
ATOM    125 MO   ???     1       8.042   5.732 -31.271
ATOM    126 FE   ???     1       7.865   5.182 -33.898
ATOM    127 FE   ???     1       5.841   6.440 -32.595
ATOM    128 FE   ???     1       6.989   8.580 -35.311
ATOM    129 FE   ???     1       5.349   7.891 -37.200
ATOM    130 FE   ???     1       6.799   6.177 -36.013
ATOM    131 FE   ???     1       4.855   7.242 -34.774
ATOM    132  C   ???     1       6.732   6.901 -34.288
ATOM    133  S   ???     1       7.668   7.717 -37.354
ATOM    134  S   ???     1       9.649   6.221 -32.968
ATOM    135  S   ???     1       4.660   5.844 -36.530
ATOM    136  S   ???     1       7.782   4.302 -35.969
ATOM    137  S   ???     1       8.574   9.635 -34.157
ATOM    138  S   ???     1       6.433   4.298 -32.345
ATOM    139  S   ???     1       4.812   9.352 -35.490
ATOM    140  S   ???     1       6.853   7.788 -31.156
ATOM    141  S   ???     1       3.694   6.777 -32.919
ATOM    142  N   ???     1       8.845   3.602 -33.464
ATOM    143  H   ???     1       9.064   3.581 -32.446
ATOM    144  N   ???     1       9.268   2.581 -34.041
ATOM    145  H   ???     1       8.992   2.684 -35.050
ATOM    146  O   ???     1       4.469   5.542 -29.477
ATOM    147  H   ???     1       5.427   5.271 -29.513
ATOM    148  H   ???     1       4.385   6.232 -30.173
ATOM    149  O   ???     1       1.066   8.390 -34.247
ATOM    150  H   ???     1       1.928   8.067 -33.885
ATOM    151  H   ???     1       0.553   8.689 -33.439
END

Fe4(trans)
REMARK Energies (QM/MM, QM+ptch) =  -17129.756264 -16290.132084
ATOM      1  H   ???     1       3.504   2.237 -29.684
ATOM      2  C   ???     1       3.922   2.083 -30.700
ATOM      3  H   ???     1       5.021   2.188 -30.635
ATOM      4  H   ???     1       3.686   1.062 -31.042
ATOM      5  N   ???     1       3.430   3.045 -31.674
ATOM      6  H   ???     1       3.941   3.941 -31.738
ATOM      7  C   ???     1       2.731   2.691 -32.767
ATOM      8  N   ???     1       1.886   1.630 -32.741
ATOM      9  H   ???     1       1.741   1.101 -31.878
```

```
ATOM     10  H   ???     1       1.664   1.166 -33.633
ATOM     11  N   ???     1       2.876   3.384 -33.894
ATOM     12  H   ???     1       3.406   4.290 -33.862
ATOM     13  H   ???     1       2.181   3.302 -34.642
ATOM     14  H   ???     1       7.276   1.565 -41.613
ATOM     15  C   ???     1       7.581   2.036 -40.674
ATOM     16  N   ???     1       8.438   3.120 -40.694
ATOM     17  C   ???     1       8.515   3.525 -39.432
ATOM     18  H   ???     1       9.092   4.365 -39.046
ATOM     19  N   ???     1       7.749   2.755 -38.613
ATOM     20  H   ???     1       7.625   2.940 -37.601
ATOM     21  C   ???     1       7.131   1.800 -39.388
ATOM     22  H   ???     1       6.438   1.073 -38.965
ATOM     23  H   ???     1       2.997   9.552 -40.121
ATOM     24  C   ???     1       3.159   8.694 -39.433
ATOM     25  H   ???     1       2.528   7.841 -39.737
ATOM     26  H   ???     1       2.939   9.004 -38.398
ATOM     27  S   ???     1       4.969   8.250 -39.578
ATOM     28  H   ???     1       6.639   8.644 -42.926
ATOM     29  C   ???     1       7.440   7.845 -42.963
ATOM     30  H   ???     1       8.014   8.035 -43.883
ATOM     31  H   ???     1       8.094   7.981 -42.083
ATOM     32  C   ???     1       6.849   6.419 -43.027
ATOM     33  O   ???     1       7.283   5.645 -43.891
ATOM     34  N   ???     1       5.934   6.118 -42.087
ATOM     35  H   ???     1       5.581   6.845 -41.443
ATOM     36  C   ???     1       5.404   4.757 -41.951
ATOM     37  H   ???     1       6.259   4.088 -42.154
ATOM     38  C   ???     1       4.924   4.512 -40.519
ATOM     39  H   ???     1       4.616   3.454 -40.442
ATOM     40  H   ???     1       5.743   4.681 -39.786
ATOM     41  O   ???     1       3.784   5.302 -40.155
ATOM     42  H   ???     1       4.132   6.166 -39.819
ATOM     43  H   ???     1       4.615   4.493 -42.669
ATOM     44  H   ???     1      13.631  11.343 -31.927
ATOM     45  C   ???     1      12.720  10.654 -31.972
ATOM     46  H   ???     1      12.875   9.949 -31.131
ATOM     47  H   ???     1      11.737  11.192 -31.789
ATOM     48  C   ???     1      12.898   9.820 -33.255
ATOM     49  O   ???     1      14.033   9.627 -33.695
ATOM     50  N   ???     1      11.809   9.209 -33.789
ATOM     51  H   ???     1      10.846   9.411 -33.486
ATOM     52  C   ???     1      11.984   8.125 -34.750
ATOM     53  H   ???     1      12.936   7.625 -34.527
ATOM     54  H   ???     1      11.148   7.421 -34.588
ATOM     55  C   ???     1      12.028   8.517 -36.240
ATOM     56  O   ???     1      13.038   8.361 -36.930
ATOM     57  N   ???     1      10.838   8.988 -36.730
ATOM     58  H   ???     1      10.008   8.880 -36.113
ATOM     59  C   ???     1      10.625   9.159 -38.153
ATOM     60  H   ???     1      11.609   9.301 -38.634
ATOM     61  H   ???     1      10.169   8.255 -38.597
ATOM     62  C   ???     1       9.697  10.295 -38.579
ATOM     63  O   ???     1       9.556  10.538 -39.784
ATOM     64  N   ???     1       9.054  10.984 -37.597
ATOM     65  H   ???     1       9.092  10.623 -36.633
ATOM     66  C   ???     1       8.105  12.045 -37.918
ATOM     67  H   ???     1       8.343  12.399 -38.931
ATOM     68  H   ???     1       7.045  11.653 -37.926
ATOM     69  C   ???     1       8.205  13.232 -36.945
ATOM     70  O   ???     1       8.459  14.374 -37.351
ATOM     71  N   ???     1       7.915  12.918 -35.659
ATOM     72  H   ???     1       7.792  11.930 -35.401
ATOM     73  C   ???     1       7.847  13.913 -34.580
ATOM     74  H   ???     1       7.005  14.598 -34.820
ATOM     75  C   ???     1       7.524  13.195 -33.264
ATOM     76  H   ???     1       8.257  12.379 -33.089
ATOM     77  H   ???     1       7.640  13.923 -32.437
ATOM     78  C   ???     1       6.101  12.617 -33.211
ATOM     79  H   ???     1       5.370  13.443 -33.333
ATOM     80  H   ???     1       5.928  11.909 -34.047
ATOM     81  C   ???     1       5.826  11.899 -31.890
ATOM     82  H   ???     1       6.469  11.001 -31.789
ATOM     83  H   ???     1       6.096  12.565 -31.045
ATOM     84  N   ???     1       4.414  11.523 -31.790
ATOM     85  H   ???     1       3.833  11.626 -32.634
ATOM     86  C   ???     1       3.957  10.577 -30.947
ATOM     87  N   ???     1       4.709  10.196 -29.906
ATOM     88  H   ???     1       5.579  10.691 -29.686
ATOM     89  H   ???     1       4.424   9.437 -29.279
ATOM     90  N   ???     1       2.733  10.045 -31.149
ATOM     91  H   ???     1       2.241  10.331 -32.006
ATOM     92  H   ???     1       2.140   9.825 -30.348
ATOM     93  H   ???     1       8.733  14.571 -34.486
ATOM     94  H   ???     1       8.403   7.457 -28.193
ATOM     95  C   ???     1       9.342   7.492 -28.768
ATOM     96  N   ???     1       9.534   6.757 -29.931
ATOM     97  C   ???     1      10.834   6.853 -30.222
ATOM     98  H   ???     1      11.334   6.391 -31.074
ATOM     99  N   ???     1      11.476   7.623 -29.308
ATOM    100  H   ???     1      12.486   7.855 -29.326
ATOM    101  C   ???     1      10.549   8.034 -28.372
ATOM    102  H   ???     1      10.829   8.696 -27.553
ATOM    103  O   ???     1      10.670   5.260 -25.626
ATOM    104  C   ???     1      10.948   4.627 -26.705
ATOM    105  O   ???     1      12.093   4.356 -27.146
ATOM    106  C   ???     1       9.732   4.242 -27.572
ATOM    107  H   ???     1       8.840   4.144 -26.927
ATOM    108  H   ???     1       9.555   5.117 -28.221
ATOM    109  C   ???     1       9.859   3.036 -28.505
ATOM    110  H   ???     1      10.851   3.054 -28.987
ATOM    111  H   ???     1       9.794   2.083 -27.943
ATOM    112  C   ???     1       8.800   3.099 -29.645
ATOM    113  O   ???     1       9.287   3.988 -30.638
ATOM    114  H   ???     1      10.003   3.057 -31.419
```

```
ATOM    115  C   ???     1       7.475   3.621 -29.071
ATOM    116  O   ???     1       7.094   4.757 -29.537
ATOM    117  O   ???     1       6.847   2.974 -28.201
ATOM    118  C   ???     1       8.514   1.732 -30.321
ATOM    119  H   ???     1       8.138   0.988 -29.599
ATOM    120  H   ???     1       7.725   1.914 -31.082
ATOM    121  C   ???     1       9.728   1.174 -31.047
ATOM    122  O   ???     1      10.437   2.090 -31.702
ATOM    123  O   ???     1      10.000  -0.026 -31.042
ATOM    124 FE   ???     1       8.025   7.526 -33.252
ATOM    125 MO   ???     1       8.058   5.573 -31.326
ATOM    126 FE   ???     1       7.817   5.076 -33.873
ATOM    127 FE   ???     1       5.866   6.152 -32.723
ATOM    128 FE   ???     1       6.652   8.422 -35.229
ATOM    129 FE   ???     1       5.604   7.362 -37.557
ATOM    130 FE   ???     1       7.031   5.827 -36.144
ATOM    131 FE   ???     1       4.863   6.655 -35.114
ATOM    132  C   ???     1       6.698   6.628 -34.409
ATOM    133  S   ???     1       7.801   7.684 -37.155
ATOM    134  S   ???     1       9.662   5.985 -33.035
ATOM    135  S   ???     1       4.992   5.296 -36.970
ATOM    136  S   ???     1       8.153   3.985 -35.709
ATOM    137  S   ???     1       8.572   9.407 -34.383
ATOM    138  S   ???     1       6.557   4.017 -32.347
ATOM    139  S   ???     1       4.528   8.681 -36.115
ATOM    140  S   ???     1       6.814   7.603 -31.281
ATOM    141  S   ???     1       3.678   6.301 -33.213
ATOM    142  N   ???     1       5.850   9.231 -33.735
ATOM    143  H   ???     1       6.561   9.684 -33.128
ATOM    144  N   ???     1       4.699   9.282 -33.229
ATOM    145  H   ???     1       4.039   8.783 -33.862
ATOM    146  O   ???     1       4.518   5.571 -29.410
ATOM    147  H   ???     1       5.459   5.257 -29.493
ATOM    148  H   ???     1       4.424   6.249 -30.112
ATOM    149  O   ???     1       1.099   8.363 -34.222
ATOM    150  H   ???     1       1.882   7.863 -33.881
ATOM    151  H   ???     1       0.602   8.665 -33.405
END

Fe2(cis)
REMARK Energies (QM/MM, QM+ptch) =  -17129.760697 -16290.131410
ATOM      1  H   ???     1       3.501   2.242 -29.688
ATOM      2  C   ???     1       3.910   2.100 -30.708
ATOM      3  H   ???     1       5.014   2.104 -30.636
ATOM      4  H   ???     1       3.588   1.123 -31.102
ATOM      5  N   ???     1       3.502   3.162 -31.616
ATOM      6  H   ???     1       3.992   4.063 -31.504
ATOM      7  C   ???     1       2.871   2.958 -32.785
ATOM      8  N   ???     1       2.007   1.916 -32.923
ATOM      9  H   ???     1       1.832   1.300 -32.128
ATOM     10  H   ???     1       1.806   1.537 -33.859
ATOM     11  N   ???     1       3.092   3.786 -33.808
ATOM     12  H   ???     1       3.648   4.656 -33.647
ATOM     13  H   ???     1       2.436   3.826 -34.594
ATOM     14  H   ???     1       7.281   1.556 -41.605
ATOM     15  C   ???     1       7.604   2.004 -40.655
ATOM     16  N   ???     1       8.573   2.988 -40.665
ATOM     17  C   ???     1       8.805   3.266 -39.386
ATOM     18  H   ???     1       9.506   4.003 -38.995
ATOM     19  N   ???     1       8.013   2.527 -38.562
ATOM     20  H   ???     1       7.928   2.719 -37.542
ATOM     21  C   ???     1       7.224   1.718 -39.355
ATOM     22  H   ???     1       6.488   1.035 -38.932
ATOM     23  H   ???     1       2.981   9.553 -40.116
ATOM     24  C   ???     1       3.112   8.696 -39.419
ATOM     25  H   ???     1       2.527   7.828 -39.770
ATOM     26  H   ???     1       2.768   9.012 -38.420
ATOM     27  S   ???     1       4.934   8.295 -39.364
ATOM     28  H   ???     1       6.632   8.637 -42.917
ATOM     29  C   ???     1       7.424   7.830 -42.945
ATOM     30  H   ???     1       7.989   7.993 -43.875
ATOM     31  H   ???     1       8.089   7.977 -42.076
ATOM     32  C   ???     1       6.823   6.410 -42.977
ATOM     33  O   ???     1       7.229   5.623 -43.840
ATOM     34  N   ???     1       5.925   6.104 -42.010
ATOM     35  H   ???     1       5.570   6.832 -41.368
ATOM     36  C   ???     1       5.374   4.747 -41.926
ATOM     37  H   ???     1       6.224   4.070 -42.127
ATOM     38  C   ???     1       4.836   4.442 -40.525
ATOM     39  H   ???     1       4.386   3.434 -40.554
ATOM     40  H   ???     1       5.667   4.379 -39.784
ATOM     41  O   ???     1       3.823   5.331 -40.066
ATOM     42  H   ???     1       4.239   6.174 -39.733
ATOM     43  H   ???     1       4.602   4.490 -42.660
ATOM     44  H   ???     1      13.632  11.343 -31.927
ATOM     45  C   ???     1      12.721  10.656 -31.970
ATOM     46  H   ???     1      12.875   9.951 -31.128
ATOM     47  H   ???     1      11.737  11.192 -31.788
ATOM     48  C   ???     1      12.898   9.819 -33.251
ATOM     49  O   ???     1      14.031   9.622 -33.694
ATOM     50  N   ???     1      11.802   9.215 -33.774
ATOM     51  H   ???     1      10.840   9.435 -33.468
ATOM     52  C   ???     1      11.955   8.132 -34.737
ATOM     53  H   ???     1      12.912   7.632 -34.537
ATOM     54  H   ???     1      11.125   7.426 -34.556
ATOM     55  C   ???     1      11.969   8.532 -36.221
ATOM     56  O   ???     1      12.979   8.418 -36.920
ATOM     57  N   ???     1      10.760   8.969 -36.708
ATOM     58  H   ???     1       9.923   8.806 -36.123
ATOM     59  C   ???     1      10.559   9.135 -38.137
ATOM     60  H   ???     1      11.554   9.236 -38.603
ATOM     61  H   ???     1      10.066   8.246 -38.571
ATOM     62  C   ???     1       9.678  10.299 -38.581
ATOM     63  O   ???     1       9.540  10.525 -39.789
ATOM     64  N   ???     1       9.068  11.028 -37.607
```

```
ATOM     65  H   ???     1       9.110  10.678 -36.643
ATOM     66  C   ???     1       8.103  12.071 -37.930
ATOM     67  H   ???     1       8.332  12.424 -38.946
ATOM     68  H   ???     1       7.045  11.662 -37.930
ATOM     69  C   ???     1       8.206  13.259 -36.958
ATOM     70  O   ???     1       8.476  14.399 -37.360
ATOM     71  N   ???     1       7.916  12.936 -35.677
ATOM     72  H   ???     1       7.735  11.951 -35.445
ATOM     73  C   ???     1       7.845  13.902 -34.572
ATOM     74  H   ???     1       6.992  14.582 -34.786
ATOM     75  C   ???     1       7.535  13.124 -33.286
ATOM     76  H   ???     1       8.317  12.356 -33.115
ATOM     77  H   ???     1       7.572  13.827 -32.432
ATOM     78  C   ???     1       6.158  12.433 -33.313
ATOM     79  H   ???     1       5.361  13.206 -33.300
ATOM     80  H   ???     1       6.024  11.847 -34.244
ATOM     81  C   ???     1       5.964  11.472 -32.139
ATOM     82  H   ???     1       6.648  10.606 -32.244
ATOM     83  H   ???     1       6.201  11.993 -31.188
ATOM     84  N   ???     1       4.569  11.022 -32.097
ATOM     85  H   ???     1       3.965  11.191 -32.916
ATOM     86  C   ???     1       3.997  10.360 -31.086
ATOM     87  N   ???     1       4.683  10.129 -29.956
ATOM     88  H   ???     1       5.629  10.505 -29.842
ATOM     89  H   ???     1       4.347   9.477 -29.240
ATOM     90  N   ???     1       2.718   9.933 -31.218
ATOM     91  H   ???     1       2.246  10.199 -32.093
ATOM     92  H   ???     1       2.113   9.912 -30.395
ATOM     93  H   ???     1       8.731  14.566 -34.480
ATOM     94  H   ???     1       8.407   7.458 -28.188
ATOM     95  C   ???     1       9.353   7.495 -28.752
ATOM     96  N   ???     1       9.565   6.746 -29.901
ATOM     97  C   ???     1      10.865   6.851 -30.186
ATOM     98  H   ???     1      11.371   6.387 -31.035
ATOM     99  N   ???     1      11.490   7.642 -29.279
ATOM    100  H   ???     1      12.496   7.886 -29.297
ATOM    101  C   ???     1      10.551   8.056 -28.355
ATOM    102  H   ???     1      10.816   8.736 -27.546
ATOM    103  O   ???     1      10.666   5.254 -25.629
ATOM    104  C   ???     1      10.946   4.621 -26.708
ATOM    105  O   ???     1      12.091   4.350 -27.148
ATOM    106  C   ???     1       9.729   4.236 -27.576
ATOM    107  H   ???     1       8.840   4.126 -26.931
ATOM    108  H   ???     1       9.546   5.117 -28.215
ATOM    109  C   ???     1       9.860   3.041 -28.523
ATOM    110  H   ???     1      10.853   3.066 -29.004
ATOM    111  H   ???     1       9.798   2.081 -27.971
ATOM    112  C   ???     1       8.800   3.108 -29.663
ATOM    113  O   ???     1       9.287   3.988 -30.668
ATOM    114  H   ???     1       9.991   3.077 -31.424
ATOM    115  C   ???     1       7.477   3.633 -29.087
ATOM    116  O   ???     1       7.093   4.764 -29.558
ATOM    117  O   ???     1       6.854   2.984 -28.213
ATOM    118  C   ???     1       8.510   1.739 -30.335
ATOM    119  H   ???     1       8.136   0.997 -29.609
ATOM    120  H   ???     1       7.718   1.915 -31.094
ATOM    121  C   ???     1       9.725   1.180 -31.062
ATOM    122  O   ???     1      10.430   2.094 -31.717
ATOM    123  O   ???     1       9.996  -0.021 -31.051
ATOM    124 FE   ???     1       5.908   6.327 -32.613
ATOM    125 MO   ???     1       8.096   5.628 -31.318
ATOM    126 FE   ???     1       7.771   5.250 -33.935
ATOM    127 FE   ???     1       8.072   7.644 -33.074
ATOM    128 FE   ???     1       4.853   7.274 -34.664
ATOM    129 FE   ???     1       6.935   6.149 -36.227
ATOM    130 FE   ???     1       5.313   7.985 -37.100
ATOM    131 FE   ???     1       6.933   8.629 -35.128
ATOM    132  C   ???     1       6.749   6.880 -34.297
ATOM    133  S   ???     1       7.639   8.104 -37.173
ATOM    134  S   ???     1       9.691   6.037 -33.024
ATOM    135  S   ???     1       4.606   5.946 -36.456
ATOM    136  S   ???     1       7.964   4.147 -35.785
ATOM    137  S   ???     1       8.574   9.622 -33.989
ATOM    138  S   ???     1       6.537   4.165 -32.424
ATOM    139  S   ???     1       4.766   9.399 -35.349
ATOM    140  S   ???     1       6.893   7.676 -31.155
ATOM    141  S   ???     1       3.734   6.717 -32.813
ATOM    142  N   ???     1       6.938   5.531 -38.048
ATOM    143  H   ???     1       6.124   4.905 -38.241
ATOM    144  N   ???     1       7.667   5.823 -39.033
ATOM    145  H   ???     1       7.290   5.366 -39.894
ATOM    146  O   ???     1       4.497   5.524 -29.484
ATOM    147  H   ???     1       5.457   5.261 -29.533
ATOM    148  H   ???     1       4.389   6.205 -30.186
ATOM    149  O   ???     1       1.092   8.387 -34.237
ATOM    150  H   ???     1       1.961   8.078 -33.879
ATOM    151  H   ???     1       0.578   8.681 -33.428
END

Fe6-cis
REMARK Energies (QM/MM, QM+ptch) =  -17129.748904 -16290.123550
ATOM      1  H   ???     1       3.503   2.240 -29.688
ATOM      2  C   ???     1       3.916   2.095 -30.708
ATOM      3  H   ???     1       5.020   2.099 -30.633
ATOM      4  H   ???     1       3.597   1.115 -31.098
ATOM      5  N   ???     1       3.511   3.150 -31.625
ATOM      6  H   ???     1       4.011   4.047 -31.529
ATOM      7  C   ???     1       2.870   2.937 -32.789
ATOM      8  N   ???     1       2.001   1.900 -32.913
ATOM      9  H   ???     1       1.833   1.286 -32.114
ATOM     10  H   ???     1       1.798   1.513 -33.846
ATOM     11  N   ???     1       3.085   3.757 -33.819
ATOM     12  H   ???     1       3.659   4.618 -33.682
ATOM     13  H   ???     1       2.437   3.782 -34.612
ATOM     14  H   ???     1       7.279   1.559 -41.618
```

ATOM	15	C	???	1	7.594	2.018	-40.682
ATOM	16	N	???	1	8.453	3.100	-40.691
ATOM	17	C	???	1	8.573	3.456	-39.417
ATOM	18	H	???	1	9.154	4.287	-39.017
ATOM	19	N	???	1	7.838	2.650	-38.604
ATOM	20	H	???	1	7.726	2.814	-37.588
ATOM	21	C	???	1	7.187	1.731	-39.393
ATOM	22	H	???	1	6.504	0.993	-38.976
ATOM	23	H	???	1	2.977	9.557	-40.117
ATOM	24	C	???	1	3.102	8.705	-39.420
ATOM	25	H	???	1	2.513	7.838	-39.764
ATOM	26	H	???	1	2.782	9.024	-38.414
ATOM	27	S	???	1	4.917	8.308	-39.408
ATOM	28	H	???	1	6.637	8.643	-42.923
ATOM	29	C	???	1	7.435	7.841	-42.957
ATOM	30	H	???	1	8.005	8.021	-43.881
ATOM	31	H	???	1	8.092	7.980	-42.081
ATOM	32	C	???	1	6.836	6.419	-43.008
ATOM	33	O	???	1	7.260	5.639	-43.871
ATOM	34	N	???	1	5.922	6.124	-42.063
ATOM	35	H	???	1	5.592	6.845	-41.403
ATOM	36	C	???	1	5.391	4.763	-41.926
ATOM	37	H	???	1	6.247	4.094	-42.127
ATOM	38	C	???	1	4.926	4.520	-40.486
ATOM	39	H	???	1	4.593	3.469	-40.414
ATOM	40	H	???	1	5.764	4.661	-39.770
ATOM	41	O	???	1	3.814	5.337	-40.092
ATOM	42	H	???	1	4.183	6.175	-39.711
ATOM	43	H	???	1	4.607	4.494	-42.655
ATOM	44	H	???	1	13.631	11.344	-31.927
ATOM	45	C	???	1	12.720	10.656	-31.972
ATOM	46	H	???	1	12.875	9.949	-31.132
ATOM	47	H	???	1	11.737	11.192	-31.789
ATOM	48	C	???	1	12.899	9.823	-33.255
ATOM	49	O	???	1	14.036	9.628	-33.692
ATOM	50	N	???	1	11.810	9.218	-33.794
ATOM	51	H	???	1	10.844	9.421	-33.493
ATOM	52	C	???	1	11.992	8.141	-34.757
ATOM	53	H	???	1	12.962	7.666	-34.553
ATOM	54	H	???	1	11.182	7.411	-34.582
ATOM	55	C	???	1	11.999	8.531	-36.243
ATOM	56	O	???	1	13.001	8.398	-36.949
ATOM	57	N	???	1	10.793	8.975	-36.724
ATOM	58	H	???	1	9.959	8.833	-36.128
ATOM	59	C	???	1	10.592	9.154	-38.150
ATOM	60	H	???	1	11.585	9.292	-38.613
ATOM	61	H	???	1	10.138	8.254	-38.602
ATOM	62	C	???	1	9.678	10.296	-38.582
ATOM	63	O	???	1	9.535	10.524	-39.790
ATOM	64	N	???	1	9.052	11.005	-37.605
ATOM	65	H	???	1	9.110	10.670	-36.635
ATOM	66	C	???	1	8.100	12.061	-37.929
ATOM	67	H	???	1	8.333	12.410	-38.946
ATOM	68	H	???	1	7.043	11.659	-37.930
ATOM	69	C	???	1	8.208	13.251	-36.959
ATOM	70	O	???	1	8.477	14.390	-37.365
ATOM	71	N	???	1	7.919	12.931	-35.678
ATOM	72	H	???	1	7.749	11.945	-35.442
ATOM	73	C	???	1	7.845	13.898	-34.573
ATOM	74	H	???	1	6.991	14.577	-34.787
ATOM	75	C	???	1	7.534	13.113	-33.290
ATOM	76	H	???	1	8.322	12.351	-33.119
ATOM	77	H	???	1	7.563	13.814	-32.433
ATOM	78	C	???	1	6.163	12.412	-33.327
ATOM	79	H	???	1	5.360	13.180	-33.309
ATOM	80	H	???	1	6.034	11.832	-34.262
ATOM	81	C	???	1	5.972	11.443	-32.159
ATOM	82	H	???	1	6.655	10.577	-32.271
ATOM	83	H	???	1	6.212	11.957	-31.206
ATOM	84	N	???	1	4.577	10.990	-32.115
ATOM	85	H	???	1	3.967	11.173	-32.925
ATOM	86	C	???	1	3.998	10.352	-31.092
ATOM	87	N	???	1	4.681	10.132	-29.957
ATOM	88	H	???	1	5.625	10.512	-29.842
ATOM	89	H	???	1	4.338	9.494	-29.232
ATOM	90	N	???	1	2.716	9.931	-31.218
ATOM	91	H	???	1	2.245	10.189	-32.097
ATOM	92	H	???	1	2.110	9.934	-30.395
ATOM	93	H	???	1	8.732	14.564	-34.480
ATOM	94	H	???	1	8.406	7.466	-28.179
ATOM	95	C	???	1	9.355	7.516	-28.727
ATOM	96	N	???	1	9.581	6.778	-29.878
ATOM	97	C	???	1	10.887	6.887	-30.144
ATOM	98	H	???	1	11.411	6.435	-30.986
ATOM	99	N	???	1	11.496	7.673	-29.224
ATOM	100	H	???	1	12.503	7.917	-29.233
ATOM	101	C	???	1	10.545	8.079	-28.310
ATOM	102	H	???	1	10.795	8.762	-27.498
ATOM	103	O	???	1	10.682	5.267	-25.600
ATOM	104	C	???	1	10.959	4.644	-26.683
ATOM	105	O	???	1	12.100	4.376	-27.134
ATOM	106	C	???	1	9.741	4.262	-27.546
ATOM	107	H	???	1	8.845	4.175	-26.907
ATOM	108	H	???	1	9.573	5.131	-28.205
ATOM	109	C	???	1	9.877	3.046	-28.458
ATOM	110	H	???	1	10.873	3.049	-28.930
ATOM	111	H	???	1	9.804	2.099	-27.889
ATOM	112	C	???	1	8.834	3.087	-29.603
ATOM	113	O	???	1	9.345	4.009	-30.600
ATOM	114	H	???	1	10.132	3.943	-33.143
ATOM	115	C	???	1	7.507	3.639	-29.079
ATOM	116	O	???	1	7.142	4.773	-29.557
ATOM	117	O	???	1	6.866	2.996	-28.216
ATOM	118	C	???	1	8.598	1.732	-30.294
ATOM	119	H	???	1	8.177	1.002	-29.581

```
ATOM    120  H   ???     1       7.851   1.892 -31.101
ATOM    121  C   ???     1       9.860   1.158 -30.959
ATOM    122  O   ???     1      10.637   2.043 -31.515
ATOM    123  O   ???     1      10.013  -0.074 -30.983
ATOM    124 FE   ???     1       8.002   7.718 -33.112
ATOM    125 MO   ???     1       8.106   5.707 -31.307
ATOM    126 FE   ???     1       7.038   8.484 -35.324
ATOM    127 FE   ???     1       5.918   6.338 -32.636
ATOM    128 FE   ???     1       8.043   5.115 -33.900
ATOM    129 FE   ???     1       6.842   6.012 -36.058
ATOM    130 FE   ???     1       5.506   7.810 -37.271
ATOM    131 FE   ???     1       4.931   7.143 -34.802
ATOM    132  C   ???     1       6.814   6.811 -34.303
ATOM    133  S   ???     1       7.727   7.676 -37.364
ATOM    134  S   ???     1       9.730   6.319 -32.955
ATOM    135  S   ???     1       4.729   5.731 -36.605
ATOM    136  S   ???     1       7.970   4.203 -35.955
ATOM    137  S   ???     1       8.551   9.629 -34.175
ATOM    138  S   ???     1       6.589   4.211 -32.397
ATOM    139  S   ???     1       4.952   9.291 -35.569
ATOM    140  S   ???     1       6.856   7.715 -31.164
ATOM    141  S   ???     1       3.764   6.713 -32.919
ATOM    142  N   ???     1       9.209   3.656 -33.551
ATOM    143  H   ???     1       9.852   1.927 -33.307
ATOM    144  N   ???     1       9.022   2.424 -33.691
ATOM    145  H   ???     1       9.992   3.379 -31.137
ATOM    146  O   ???     1       4.526   5.525 -29.486
ATOM    147  H   ???     1       5.483   5.254 -29.540
ATOM    148  H   ???     1       4.426   6.208 -30.188
ATOM    149  O   ???     1       1.092   8.389 -34.233
ATOM    150  H   ???     1       1.947   8.051 -33.868
ATOM    151  H   ???     1       0.573   8.683 -33.426
END
```
